# Diagnostic Efficacy and Clinical Aspects of Panoramic‐View Versus Forward‐View Capsule Endoscopy for Suspected Small Bowel Bleeding: A Systematic Review and Meta‐Analysis

**DOI:** 10.1002/hsr2.72095

**Published:** 2026-05-03

**Authors:** Ilya Saadati, Reza Elahi, Mehdi Azizmohammad Looha, Seyed Amir Ahmad Safavi‐Naini, Shabnam Shahrokh

**Affiliations:** ^1^ Basic and Molecular Epidemiology of Gastrointestinal Disorders Research Centre, Research Institute for Gastroenterology and Liver Diseases Shahid Beheshti University of Medical Sciences Tehran Iran; ^2^ Department of Radiology Zanjan University of Medical Sciences Zanjan Iran

**Keywords:** CapsoCam, capsule endoscopy, diagnostic yield, forward‐view CE, systematic review

## Abstract

**Background and Aims:**

CapsoCam provides a 360° panoramic‐view in capsule endoscopy (CE). This study aimed to conduct the initial systematic review and meta‐analysis on the diagnostic efficacy and clinical aspects of CapsoCam in patients with Suspected small bowel bleeding (SSBB), potentially comparing it to forward‐view CE.

**Methods:**

A systematic search of PubMed, Embase, and Scopus databases was performed for human studies reporting outcomes of interest up to January 2025. Titles and abstracts were screened, and full‐texts were selected based on predefined inclusion criteria by two reviewers. The quality assessment used Joanna Briggs Institute checklist. Meta‐analysis used a random‐effects model with a 95% confidence interval (CI). Publication bias was evaluated using Egger and Begg's tests. A leave‐one‐out sensitivity analysis was performed.

**Results:**

Five studies met the inclusion criteria. The meta‐analysis revealed no statistically significant difference in diagnostic yield between the two CE types in cases of SSBB (*p* = 0.89, 95% CI: −0.08 to 0.09). Egger's test and Begg's test showed no significant publication bias. Comparative analysis between panoramic and forward‐view CEs demonstrated similar performance for small bowel transit time (*p* = 0.58, 95% CI: −0.33 to 0.59), although evaluation time was significantly longer with CapsoCam (*p* = 0.001, 95% CI: 0.42 to 1.60). The risk difference in completion rates was not statistically significant (*p* = 0.91, 95% CI: −0.13 to 0.15). Subgroup analyses showed no significant differences in diagnostic yield or other outcomes between CapsoCam PLUS and SV‐1, or by study design.

**Conclusion:**

Our analysis suggests that CapsoCam demonstrates comparable performance to forward‐view CE in terms of diagnostic reliability, clinical efficacy, and safety profile, except for reading time. However, the limited number of studies underscores the need for further research to validate CapsoCam's usability in routine clinical practice for SSBB patients compared to forward‐view CE.

**Abbreviations**
CE: capsule endoscopy
CI: confidence interval
DY: diagnostic yield
RD: risk difference
SB: small bowel
SD: standard deviation
SMD: standardized mean difference
SSBB: suspected small bowel bleeding

## 1 Background

Suspected small bowel bleeding (SSBB) refers to bleeding within the gastrointestinal tract that remains undiagnosed despite a thorough evaluation using upper endoscopy and colonoscopy. It may present as overt bleeding, such as visible blood in the stool, or as chronic iron deficiency anemia caused by occult blood loss. Diagnosing SSBB presents a significant clinical challenge, often requiring advanced diagnostic techniques and careful patient management [1, 2]. Capsule endoscopy (CE) is a groundbreaking non‐invasive diagnostic technology primarily utilized to investigate the small bowel (SB) in patients presenting with gastrointestinal disorders. Since its introduction, CE has become the first‐line diagnostic modality for SSBB, providing a less invasive alternative to traditional endoscopic techniques and facilitating the visualization of areas inaccessible by conventional methods [2, 3].

Conventional CE systems are predominantly forward‐viewing, incorporating a single camera positioned at the head of the capsule. In conjunction with a light source and an image sensor, this camera captures images along the capsule's trajectory, providing an angle of view ranging from 140° to 170°. While effective, this limited field of view may result in missed mucosal lesions, particularly in regions not directly visualized by the camera, potentially compromising diagnostic accuracy [4]. Recognizing these limitations, panoramic‐view CE systems have been developed to broaden the angle of view and reduce the miss rate, aiming to enhance diagnostic precision and improve clinical outcomes [3].

One of the most notable advancements in this domain is the CapsoCam (CapsoVision, Cupertino, California, USA), a CE system that utilizes a unique forward/panoramic‐view configuration. Unlike traditional forward‐view capsules, CapsoCam has four cameras strategically positioned medially within the capsule, each providing a 90° viewing angle. This design enables a 360° panoramic‐view of the SB mucosa, offering comprehensive visualization and reducing the likelihood of undetected lesions [5]. CapsoCam further incorporates advanced technological features, including an adaptive frame rate ranging from 3 to 5 frames per second per camera, which is adjusted according to the transit time through the gastrointestinal tract. This functionality optimizes image acquisition while conserving battery life, which exceeds 15 h, enhancing the procedure's efficiency [2].

A distinctive feature of CapsoCam is its internal image storage capability. Unlike conventional CE systems, which rely on real‐time data transmission to an external recorder worn by the patient, CapsoCam stores all captured images internally on a microchip. This eliminates the need for external recording devices, potentially increasing patient comfort and satisfaction. Furthermore, the internal storage design enables self‐administration of the procedure at home, expanding its accessibility and convenience [4]. However, capsule retrieval from stool is required to access the stored data, introducing a unique logistical consideration that may influence patient compliance and usability [3].

The potential advantages of panoramic‐view CE, including a wider field of view, adaptive frame rate, motion‐activated operation, and internal data storage, suggest significant advancements over traditional forward‐view CE systems. These features are anticipated to improve diagnostic efficacy, enhance visualization of SB mucosa, and increase patient satisfaction by eliminating the need for cumbersome external equipment. Despite these promising advancements, no consensus has been made on the clinical benefits of panoramic‐view CE regarding diagnostic accuracy, patient outcomes, and cost‐effectiveness relative to conventional forward‐view CE systems, which warrant rigorous empirical evaluation.

This systematic review and meta‐analysis aim to systematically review the evidence on panoramic‐view CE's diagnostic efficacy and clinical utility compared to forward‐view CE. By focusing on their respective performances in evaluating SSBB, this study seeks to elucidate these technologies’ relative strengths and limitations, providing insights into their clinical implications and informing future research and practice.

## 2 Methods

This systematic review is registered with PROSPERO (International Prospective Register of Systematic Reviews) under the registration code CRD42022381504. The study design and reporting were conducted in accordance with PRISMA 2020 (Preferred Reporting Items for Systematic Reviews and Meta‐Analyses) guidelines [6], as recommended by the EQUATOR Network (Enhancing the QUAlity and Transparency Of health Research) [7].

### 2.1 Definition of Terms

**Diagnostic Yield (DY):** In this review, DY was generally defined as the proportion of patients with positive CE findings divided by the total number of patients examined. Because DY represented the primary outcome of our review, we adhered to the definition provided in each included article to respect methodological differences. The included studies did not consistently apply a uniform definition of DY, and variations were observed across several dimensions: the lesion classification system (P0, P1, P2) [8], the anatomical location of lesions considered (gastric, SB, colon), and the denominator used for the DY fraction (all recruited patients *vs.* only those with a complete CE procedure). A study‐by‐study description is provided below:

A study defined DY as the proportion of patients with SB lesions detected by CE. The denominator included all recruited patients, and no specification of lesion class (P0, P1, P2) was provided [9].

Another study calculated DY by including only patients who completed the CE procedure. Both P1 and P2 lesions were considered positive findings, and only SB lesions were included in the definition [2].

Two other studies included all recruited patients in the denominator. While these studies did not explicitly restrict analysis to SB lesions, the patient cohort of each consisted of individuals investigated for SSBB. In both of them DY was reported separately for P1 and P2 lesions, and in our review, we extracted the P2‐based definition [10, 11].

There was also another study which considered all recruited patients in the denominator. The study did not specify lesion class but reported DY separately for gastric, SB, and colonic lesions. For the purpose of this review, we extracted the SB–based DY definition [12].

**Completion Rate:** Refers to the success rate of the CE procedure, encompassing all stages from capsule ingestion to data interpretation.

**Technical Fault:** Describes a procedural failure in a patient despite successful capsule ingestion, expulsion, and retrieval (if required). This includes errors during data recording, exporting (in CapsoCam systems), or transmission (in forward‐view CE).

**Capsule Retention:** Defined as the persistence of a capsule within the gastrointestinal tract for at least 2 weeks. This occurs in approximately 2% of patients undergoing CE [13]. Common causes include Crohn's disease, obstructive tumors, and diaphragm disease associated with adverse effects of nonsteroidal anti‐inflammatory drugs [14].

**Incomplete Capsule Transit:** Occurs when the capsule fails to reach the cecum within the allotted recording time, typically due to delayed transit and prolonged retention in a single intestinal segment for more than 2 h, leading to battery depletion [15].

### 2.2 Research Questions

The primary objective of this systematic review is to address the question: Does panoramic‐view CE provide superior clinical efficacy, technical performance, and safety compared to forward‐view CEs? To explore this question, the review is guided by the following sub‐questions:

How does panoramic‐view CE's diagnostic accuracy compare to traditional forward‐view CE?

Does panoramic‐view CE result in shorter gastric and SB transit times in patients with SSBB?

Is panoramic‐view CE more time‐efficient in interpretation than forward‐view CE?

Does panoramic‐view CE achieve higher completion rates and lower instances of incomplete transit in patient examinations?

Which modality—panoramic‐view or forward‐view CE—has lower rates of capsule retention, technical malfunctions, and inadequate cleaning, factors that may compromise the procedure's usability?

### 2.3 Search Strategy

This review evaluates the clinical efficacy, technical performance, and safety profile of CapsoCam, a novel panoramic‐view CE, compared to traditional forward‐view CEs. The analysis is restricted to studies involving capsule administration in patients with SSBB and includes peer‐reviewed journal articles and conference papers published in English.

A comprehensive literature search was conducted to identify relevant studies. Searches were performed in PubMed, Embase, and Scopus between January 2025. A detailed search strategy was developed using MeSH (Medical Subject Headings) and non‐MeSH terms to maximize inclusivity. The search focused on four key themes: (1) terms related to CE as the central topic, (2) terms describing the panoramic‐view capability of CE, (3) terms specific to forward‐view CE as the comparator, and (4) terms associated with the pathology of interest, SSBB. To ensure thorough coverage, reference lists of included articles were manually reviewed, and PubMed's “similar articles” feature was utilized. The final search queries for each database are presented in Table S1.

Duplicate publications were identified and removed using EndNote (EndNote X9.3.3) [16] and manual cross‐checking by reviewers. Two independent reviewers (I.S. and S.A.A.S.N.) screened the titles and abstracts to exclude irrelevant citations. Full texts of potentially eligible studies were then assessed independently for inclusion. Any discrepancies were resolved through discussion or consulting a third reviewer when necessary.

### 2.4 Study Selection and Eligibility Criteria

**Type of Publication**: Original research articles and conference abstracts published in English, available as full papers or abstracts.

**Study Population**: Studies employing panoramic‐view and forward‐view CE, either within the same patient cohort or across different cohorts, to evaluate SSBB.

**Outcome Measures**: Studies report the DY of panoramic‐view and forward‐view capsules in patients with SSBB.

**Exclusion Criteria** Studies were excluded if they were review articles, animal studies, in vivo or in vitro experiments, case reports, case series with fewer than three cases, news articles, modeling studies, practice guidelines, clinical opinions, commentaries, papers without full texts, or those lacking relevant data.

### 2.5 Outcomes Measured

The primary outcome was CE's diagnostic effectiveness in detecting pathological lesions, which was assessed using DY to measure its clinical utility. Secondary outcomes included technical performance metrics, such as SB transit time, gastric transit time, and reading time. Additionally, procedural success and safety measures were evaluated, including completion rate, retention rate, incomplete transit rate, technical failure rate, and insufficient cleaning rate.

### 2.6 Data Extraction

Two authors (I.S. and S.A.A.S.N.) independently extracted data using a predefined Microsoft Excel template. The extracted data included the first author's name, year of publication, study location, study design, the total number of patients enrolled, number of patients assigned to forward or panoramic CE, type of capsule used, and comparison made in the study, DY of each CE type or the number of patients with clinically significant lesions for DY calculation, specifying whether DY was for overt or occult bleeding, SB, and gastric transit times for each CE type, evaluation time required for each CE type, the completion rate for each CE type, the retention rate for each CE type, number of technical faults associated with each CE type, number of patients experiencing incomplete transit for each CE type., insufficient bowel cleaning rates for each capsule type, and key findings of the study.

### 2.7 Risk of Bias Assessment

The methodological validity of the selected articles was evaluated using the Joanna Briggs Institute (JBI) Diagnostic Test Accuracy Review Instrument's Critical Appraisal Checklist [17] (Table S2). Two independent reviewers (I.S. and S.A.A.S.) conducted the appraisal, resolving any disagreements through discussion.

### 2.8 Statistical Method and Meta‐Analysis

A comprehensive statistical and meta‐analytical framework was employed to analyze the data. The process began with data preprocessing to ensure dataset integrity. Incomplete cases were excluded, and a continuity correction value of 0.5 was applied when necessary to address zero‐event cells. For proportion‐based analyses, adjusted proportions were computed for the panoramic (experimental) and forward‐view (control) groups, and the differences in proportions with their corresponding variances were calculated. For continuous outcomes, standardized mean differences (SMDs) and their variances were derived using reported means, standard deviations (SDs), and sample sizes.

The meta‐analysis was conducted using the meta and metafor R packages [18, 19]. Random‐effects models were fitted using the restricted maximum‐likelihood (REML) estimator [20]. For binary outcomes, we used the metabin function in the meta package to obtain pooled risk differences (RDs) with 95% confidence intervals (CIs). For continuous outcomes, the metacont function (meta) and the escalc/rma functions (metafor) were applied to estimate pooled SMDs with 95% CIs. Forest plots were generated to display individual study results and pooled estimates, clearly distinguishing between panoramic and forward‐view CE.

Heterogeneity across studies was evaluated using Cochran's *Q* test [21] with its associated *p* value, the *I*² statistic to quantify the percentage of variability due to heterogeneity rather than chance [22], and τ² to estimate between‐study variance [23]. Publication bias was assessed through visual inspection of funnel plots and formally tested using Egger's regression test for funnel plot asymmetry [24] and Begg's rank correlation test [25]. Galbraith plots were additionally used to explore residual heterogeneity and identify potential outliers [26].

Sensitivity analyses were performed using a leave‐one‐out approach, in which each study was excluded in turn and the meta‐analysis repeated. This allowed identification of influential studies and evaluation of the robustness of the pooled estimates. All statistical tests were two‐sided, and an a priori significance level of α = 0.05 was adopted. *p* values are reported in accordance with international standards: *p* < 0.001, or to three decimal places when between 0.001 and 0.01, to two decimal places when between 0.01 and 0.99, and as *p* > 0.99 when applicable.

The primary analyses, consisting of the overall pooled estimates for both binary and continuous outcomes, were prespecified before conducting the review. These analyses were designed to provide the main effect estimates comparing panoramic‐ versus forward‐view CE. In addition, we performed exploratory subgroup analyses to investigate potential sources of heterogeneity. Specifically, we stratified studies by (i) study design (e.g., randomized controlled trials, and retrospective studies) and (ii) type of panoramic capsule used (e.g., CapsoCam SV‐1, CapsoCam Plus, CapsoCam a). Subgroup analyses were conducted using the byvar option in the meta package, which generates subgroup‐specific pooled estimates under a random‐effects model without computing an overall combined effect. To assess differences between subgroups, we applied a test for subgroup differences (moderator analysis) using the rma function from metafor, yielding a Q statistic (QM) and corresponding *p* value. Because these subgroup analyses were not prespecified in the study protocol, they should be considered exploratory and their results interpreted with caution as hypothesis‐generating rather than confirmatory.

All analyses were conducted using R software [27], version 4.5.0 (R Foundation for Statistical Computing, Vienna, Austria), employing the meta and metafor packages for meta‐analyses and the ggplot2 and grid packages for graphical output. Reporting of statistical methods follows the SAMPL (Statistical Analyses and Methods in the Published Literature) guidelines [28] to ensure transparency and reproducibility.

## 3 Result

### 3.1 Study Characteristics

After a thorough evaluation, only five studies met all the predetermined inclusion criteria and were included for detailed analysis in the systematic review [2, 9, 10, 11, 12]. The study selection and exclusion process are clearly illustrated in the Graphical Abstract section. Three of the included studies were published as peer‐reviewed articles [2, 9, 11], while the remaining two were conference abstracts [10, 12]. Two of the studies originated from East Asia, with one from Japan and one from Taiwan [10, 11]. The remaining three studies were conducted in European centers, specifically in Germany, the United Kingdom, and France [2, 9, 12]. The risk of bias assessment results is available in Table 3. Three studies were rated as having a score of 3, while two studies were rated as having a score of 4. All studies used CapsoCam as the panoramic‐view CE, four utilized the PillCam (Medtronic, Minneapolis) as the forward‐view CE, and one employed the EndoCapsule (Olympus, Japan). With respect to CapsoCam generations, two studies utilized CapsoCam SV‐1, two studies employed CapsoCam Plus (the newer generation), and one study did not specify the generation of the capsule used. Table 1 provides detailed descriptions of the studies.

**Table 1:** Characteristics of the included studies.

Type of SSBB (occult, overt or both)	Occult	Both	Both	Both	Overt
Time period	May 2013 and December 2015	April 2011 and June 2012	March 2014 and October 2023	NM	NM
Country	Germany	France	Japan	Taiwan	United Kingdom
Forward‐view CE type	PillCam SB 3	PillCam SB 2	PillCam SB 3	Olympus EndoCapsule 10	PillCam SB 3
Panoramic‐view CE type	CapsoCam SV‐1	CapsoCam SV‐1	CapsoCam Plus	CapsoCam Plus	CapsoCam^a^
Number of populations (male percentage)	153 (56.86%)	73 (36.99%)	66 (60.61%)	16 (43.75%)	94 (57.45%)
Study design	Prospective randomized multicenter trial	Prospective comparative Study	Retrospective with propensity score matching	Prospective randomized controlled study	NM
Year of publication	2018	2014	2024	2023	2019
Study	Lilli L. Zwinger et al.	Mathieu Pioche et al.	Hirata et al.	SUNG et al.	Zammit et al.

^a^ The specific generation of CapsoCam was not reported. NM; not mentioned.

### 3.2 Participant Characteristics

A total of 402 patients were enrolled across the selected studies, with the sample sizes from 16 to 153 participants, with male gender rates ranging from 37% to 61%. All selected articles reported either the mean or median age of participants and included information on sex distribution. One study focused on patients with occult SSBB [9], while another specifically examined overt SSBB [12]. The remaining studies included both patient groups.

### 3.3 Diagnostic Yield

**Figure 1:**
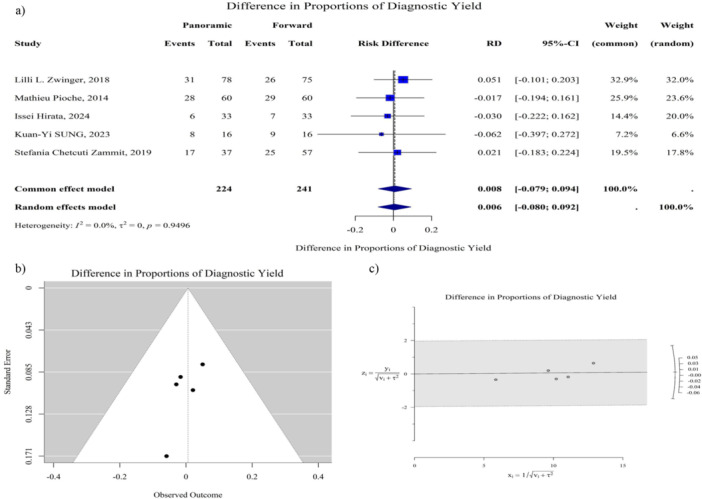
Comparative analysis of diagnostic yield between panoramic‐view and forward‐view capsule endoscopy in patients with suspected small bowel bleeding. (a) Forest plot showing both common‐effect and random‐effect model results for diagnostic yield across included studies. Heterogeneity is reported as *I*² (proportion of variation due to true differences rather than chance), τ² (between‐study variance), and *p* value. (b) Funnel plot displaying standard error versus observed outcome for diagnostic yield to assess potential publication bias. (c) Galbraith plot evaluating heterogeneity for diagnostic yield, where y **=** observed effect/√ (v + τ²) and x = 1/√ (v + τ²), with v being the variance of the study effect and τ² the between‐study variance. Diagnostic yield is generally defined as the proportion of patients with positive capsule endoscopy findings divided by the total number of patients examined. CI = confidence interval, RD = risk difference.

The pooled RD between panoramic‐view CE and forward‐view CE was 0.01 (95% CI: −0.08 to 0.09, *p* = 0.89), indicating that the DY of the two CE types is comparable. As shown in Figure 1, the heterogeneity among the studies was minimal (*I*² = 0.0%, *p* = 0.95). Both Egger and Begg's tests indicated no statistically significant publication bias. Additionally, the leave‐one‐out sensitivity analysis showed that excluding any individual study had minimal impact on the overall pooled risk difference. The estimate ranged from −0.01 to 0.01, with no substantial changes in the direction or magnitude of the effect (Figure S1).

### 3.4 Transit Time

SB transit time was reported in four studies (80%) [2, 9, 10, 11]. The pooled standardized mean difference (SMD) was 0.13 (95% CI: −0.33 to 0.59; *p* = 0.58), indicating no statistically significant difference between the two CE types. Heterogeneity was high (*I*² = 77.9%).

Gastric transit time was reported in three studies [2, 10, 11]. The pooled SMD was –0.16 (95% CI: −0.42 to 0.11; *p* = 0.25), again showing no significant difference. Heterogeneity was negligible (*I*² = 0.0%).

**Figure 2:**
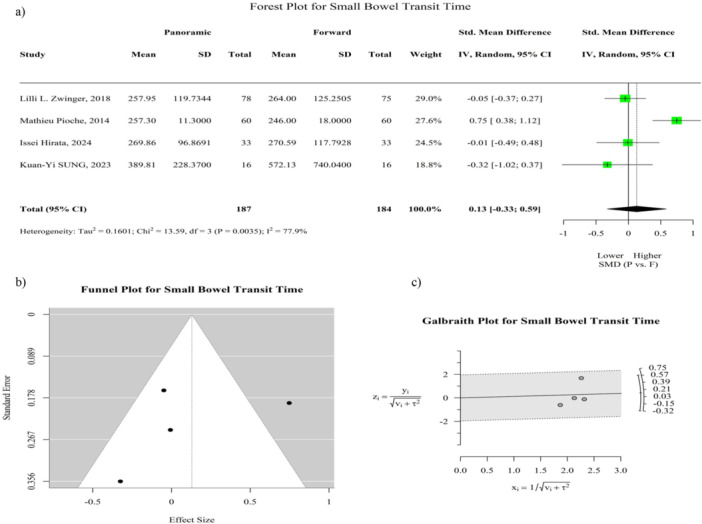
Small bowel transit time comparison between panoramic‐view and forward‐view capsule endoscopy. (a) Forest plot reporting common‐effect and random‐effect models for small bowel transit time. Heterogeneity is reported as *I*² (proportion of variation due to true differences rather than chance), τ² (between‐study variance), and *p* value. (b) Funnel plot of standard error versus observed small bowel transit time to assess potential publication bias. (c) Galbraith plot for small bowel transit time: y = observed effect/√(v + τ²), x = 1/√(v + τ²), with v being the study variance and τ² the between‐study variance. Small bowel transit time is defined as the duration from capsule entry into the duodenum to entry into the cecum. CI = confidence interval, SD = standard deviation, SMD = standardized mean difference.

As shown by the visual assessment of the funnel plots in Figures 2 and S2, there was no statistically significant publication bias among the studies for SB and gastric transit times. The sensitivity analysis showed that excluding individual studies had minimal impact on the overall estimates, as the effect size remained stable and the confidence interval still included zero for both SB and gastric transit time (Figures S3, S4).

### 3.5 Reading Time

**Figure 3:**
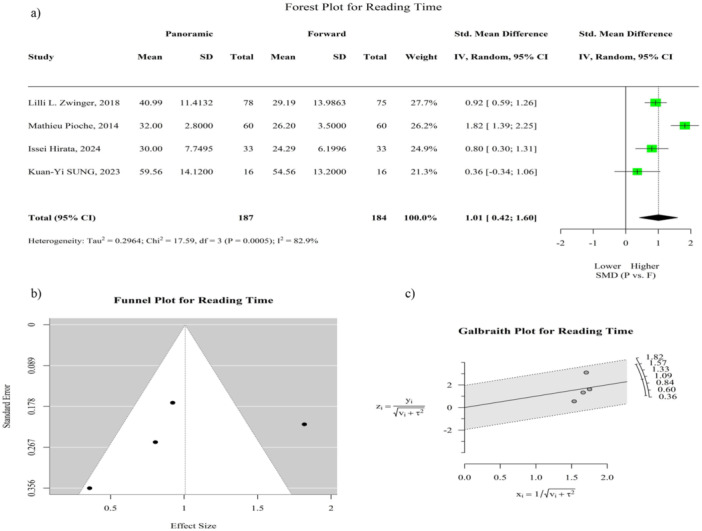
Reading time comparison between panoramic‐view and forward‐view capsule endoscopy. (a) Forest plot of standardized mean differences in reading time across studies. (b) Funnel plot of standard error versus observed reading time outcome to assess potential publication bias. (c) Galbraith plot for reading time: y = observed effect/√(v + τ²), x = 1/√(v + τ²), with v being the study variance and τ² the between‐study variance. Reading time is defined as the total duration required for reviewing capsule images by clinicians. CI = confidence interval, SD = standard deviation, SMD = standardized mean difference.

Reading time was mentioned in four studies [2, 9, 10, 11]. The *Q* test revealed significant statistical heterogeneity across the included studies (*I*² = 82.9%; *p* = 0.001). The pooled SMD was 1.01 (95% CI: 0.42–1.60; *p* < 0.001), indicating that the reading time for panoramic‐view CE was statistically significantly longer than that for forward‐view CE. This difference is clearly illustrated in the forest plot shown in Figure 3. Egger's and Begg's tests did not identify statistically significant publication bias. In addition, sensitivity analyses demonstrated the robustness of this finding, as the removal of any single study did not substantially alter the effect size, and the confidence intervals consistently remained above zero in all scenarios (Figure S5).

### 3.6 Rates of Completion, Technical Fault, Retention, and Incomplete Transit

Four studies provided sufficient data to perform a meta‐analysis on completion, technical fault, retention, and incomplete transit rates [2, 9, 10, 11]. The methodological validity of all included studies was evaluated using the described risk of bias method to identify potential biases (Table 2). Based on Begg's and Egger's tests, publication bias was not statistically significant. As demonstrated in Figures S6–S9, the sensitivity analysis confirmed that no single study had unduly influenced our data.

**Table 2:** Results of the meta‐analysis.

	Number of studies	Estimated pooled effect measure	Confidence interval (95%)	*p* value	I^2^ (*Q* test *p*‐value)	Begg *p* value	Egger *p* value
Diagnostic yield^a^	5	0.01	[−0.08, 0.09]	0.89	0.0% (0.95)	0.483	0.21
Small bowel transit time^b^	4	0.13	[−0.33, 0.59]	0.58	77.9% (0.004)	> 0.99	0.70
Gastric transit time^b^	3	−0.16	[−0.42, 0.11]	0.25	0.0% (0.70)	0.602	0.44
Reading time^a^	4	1.01	[0.42, 1.60]	0.001	82.9% (0.001)	0.497	0.67
Completion rate^a^	4	0.01	[−0.13, 0.15]	0.91	74.4% (0.008)	0.750	0.56
Technical fault rate^a^	4	0.01	[−0.02, 0.04]	0.39	73.5% (0.01)	0.750	0.31
Retention rate^a^	4	0.01	[−0.01, 0.03]	0.50	0.00% (0.59)	0.750	0.73
Incomplete transit rate^a^	4	−0.05	[−0.15, 0.05]	0.32	62.7% (0.05)	0.750	0.37
Insufficient preparation rate^a^	4	0.00	[−0.03, 0.02]	0.71	0.0% (0.86)	0.750	0.63

*Note:* In a study reporting median and IQR, approximation was used for meta‐analysis [29].

^a^ Effect measure calculated using risk difference.

^b^ Effect measure calculated using standardized mean difference.

**Table 3:** Results of quality assessment using the Joanna Briggs Institute checklist. Each study was evaluated based on ten questions, with responses categorized as Yes, No, Unknown, or Not Applicable. The quality score for each study was determined by the total number of “Yes” responses.

Year	Author	Country	Quality
2018	Zwinger	Germany	3
2014	Pioche	France	4
2024	Hirata	Japan	3
2023	SUNG	Taiwan	3
2019	Zammit	UK	4

**Figure 4:**
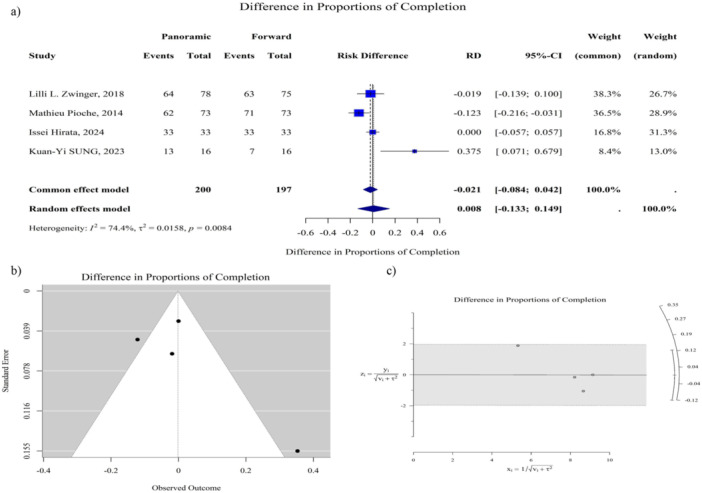
Completion rate comparison between panoramic‐view and forward‐view capsule endoscopy. (a) Forest plot with common‐effect and random‐effect model results. Heterogeneity reported as I², τ², and *p* value. (b) Funnel plot of standard error versus observed completion outcome to assess potential publication bias. (c) Galbraith plot for completion rate, y = observed effect/√ (v + τ²), x = 1/√ (v + τ²), with v being study variance and τ² between‐study variance. Completion rate refers to the success rate of the capsule endoscopy procedure, encompassing all stages from capsule ingestion to data interpretation. CI = confidence interval, RD = risk difference.

For completion rate, the pooled RD was 0.01 (95% CI: –0.13 to 0.15; *p* = 0.91), indicating no statistically significant difference between panoramic‐view and forward‐view CE, although heterogeneity across the studies was substantial (*I*² = 74.4%), as shown in Figure 4. For technical faults, the pooled RD was 0.01 (95% CI: −0.02 to 0.04; *p* = 0.39), with significant heterogeneity detected (*I*² = 73.5%; *p* = 0.01), as depicted in Figure S10. For retention rates, the pooled RD was 0.01 (95% CI: –0.01 to 0.03; *p* = 0.50), showing no statistically significant difference between the two CE types, and heterogeneity was negligible (*I*² = 0.0%), as shown in Figure S11. Finally, for incomplete transit, the pooled RD was –0.05 (95% CI: −0.15 to 0.05; *p* = 0.32), again suggesting no statistically significant difference between the two modalities. In this analysis, moderate heterogeneity was observed (*I*² = 62.7%), as depicted in Figure S12.

### 3.7 Subgroup Analysis

#### 3.7.1 Panoramic Capsule Type

Stratification by capsule type showed no significant differences in DY between panoramic and forward‐view CE. The pooled RD was –0.04 (95% CI: −0.21 to 0.14; *p* = 0.22) for *CapsoCam PLUS* and 0.02 (95% CI: −0.40 to 0.45; *p* = 0.63) for *CapsoCam SV‐1*. Completion, retention, incomplete transit, and technical fault rates were also non‐significant across subgroups. For continuous outcomes, SB transit times (SMD = –0.11; 95% CI: −2.00 to 1.78; *p* = 0.60 for *PLUS*, and 0.34; 95% CI: −4.72 to 5.40; *p* = 0.55 for *SV‐1*) and gastric transit times (SMD = –0.03; 95% CI: −0.21 to 0.15; *p* = 0.27 for *PLUS*) showed no differences. Reading time was numerically longer with panoramic CE but not statistically significant (Figures S13–S21, Table S3).

#### 3.7.2 Study Design

By study design, DY remained comparable across subgroups (RD = 0.01; 95% CI: −0.11 to 0.14; *p* = 0.69 in RCTs vs –0.01; 95% CI: −0.33 to 0.32; *p* = 0.85 in retrospective studies). Other binary outcomes, including completion, retention, and incomplete transit, also showed no subgroup effects. In RCTs, SMDs for SB transit (0.16; 95% CI: −1.21 to 1.53; *p* = 0.66), gastric transit (–0.22; 95% CI: −1.30 to 0.87; *p* = 0.24), and reading time (1.06; 95% CI: −0.74 to 2.87; *p* = 0.13) were not statistically significant. Retrospective studies included too few data for pooling. Thus, results were consistent regardless of study design (Figures S22–S30, Table S4).

## 4 Discussion

SB CE is the preferred method for evaluating SSBB due to its non‐invasive nature and proven efficacy [2]. However, there is no consensus on whether CapsoCam or forward‐view CE offers superior diagnostic performance. This systematic review synthesizes the available comparative evidence and demonstrates that the two systems perform similarly across key outcomes, including DY, completion rate, safety, and technical reliability. The only consistent difference identified was a longer reading time with panoramic CE, likely reflecting the increased image volume from 360° visualization.

DY was comparable between panoramic and forward‐view capsules. Our meta‐analysis confirmed similar effectiveness in detecting lesions associated with SSBB. This suggests that, despite theoretical advantages of wider viewing angles, panoramic CE does not currently offer superior diagnostic performance.

Procedural outcomes were also broadly similar. Small bowel and gastric transit times did not differ significantly between capsule types, although substantial heterogeneity was observed in small bowel transit times, limiting confidence in this finding. Reading time was consistently longer for panoramic CE, likely reflecting the larger number of images generated by 360° visualization. While not prohibitive, this may reduce efficiency in clinical practice, and future research should evaluate strategies such as artificial intelligent‐assisted review to mitigate this burden.

Completion rates, technical reliability, retention, and incomplete transit rates showed no significant differences. Device‐related faults, such as data retrieval failures in panoramic capsules or image transmission issues in forward‐view devices, occurred infrequently. Retention rates were low and comparable, consistent with reported CE retention rates in the broader literature [30]. Importantly, incomplete transit rates did not differ, indicating that both technologies achieve similar procedural success in reaching the cecum. These findings collectively suggest that panoramic CE does not compromise safety or technical performance.

Two included studies [10, 11] reported that total procedure time was longer for panoramic‐view CE, reflecting the need to retrieve the capsule after passage for data analysis. Panoramic CE also demonstrated superior detection of the ampulla of Vater in both studies, while only one study reported improved visualization of the Z‐line [10]. Patient acceptance was generally favorable across studies. Hirata et al. found similar levels of swallowing difficulty and discomfort between capsule types, with a slight preference for CapsoCam [11], while Zwinger et al. reported high satisfaction with the panoramic system, supporting its potential acceptability in ambulatory settings [9].

Safety outcomes were reassuring, with only two severe adverse events among 402 patients across two studies [9, 10]. These included a mechanical ileus caused by a gastrointestinal stromal tumor obstructing capsule passage and pathological retention due to jejunal stenosis. Both were managed successfully. Overall, serious adverse events occurred in < 1% of patients, underscoring the safety profile of both capsule types.

Subgroup analyses did not reveal clear differences between CapsoCam PLUS and SV‐1, and the available data do not suggest that the newer PLUS model is superior in diagnostic or technical outcomes. Similarly, stratification by study design showed generally consistent results across RCTs and retrospective studies, although caution is warranted in interpretation given the limited number of studies.

### 4.1 Limitations

This review has several limitations. First, the number of available studies was small, and some outcomes (e.g., patient acceptance, Z‐line detection, follow‐up) were reported by only one or two studies, limiting generalizability. Second, definitions of DY varied, including differences in lesion classification (P0–P2 in Saurin system), anatomical sites analyzed, and denominators used. While we adhered to each study's reported definition, these inconsistencies may reduce comparability. Third, heterogeneity was moderate to high for some outcomes, particularly small bowel transit and reading time, reducing confidence in pooled estimates. Fourth, several included reports were conference abstracts without full peer‐reviewed manuscripts, raising concerns about reporting bias and incomplete methodological detail. Fifth, no quality assessment tool specifically addresses DY as the primary outcome; the Joanna Briggs Institute checklist provided only partial applicability. All included studies compared panoramic CE to forward‐view CE; none included other diagnostic modalities such as device‐assisted enteroscopy or imaging, limiting the ability to contextualize findings across all available approaches.

## 5 Conclusion

Our results demonstrate that CapsoCam and forward‐view CE have comparable diagnostic reliability in patients with SSBB. Except for the reading time, which was statistically significantly longer with CapsoCam, all other measures of clinical efficacy and safety were similar between the two CE modalities. Given its comparable diagnostic performance in SSBB patients, further studies are needed to evaluate whether the use of CapsoCam in routine clinical practice is justified. Large‐scale studies are essential to further compare the applicability and diagnostic efficacy of CapsoCam versus forward‐view CE in diagnosing SSBB.

## Author Contributions

**Ilya Saadati:** conceptualization, data curation, writing – original draft, visualization, finalization. **Reza Elahi:** conceptualization, writing – review and editing. **Mehdi Azizmohammad Looha:** methodology, formal analysis, writing, review and editing, visualization. **Seyed Amir Ahmad Safavi‐Naini:** conceptualization, data curation, validation, supervision. **Shabnam Shahrokh:** validation, project administration. All authors read and approved the final manuscript.

## Funding

All authors confirm that this study did not receive any financial support, grants, or external funding. No financial relationships or supporting sources were involved in the study design, data collection, analysis, interpretation of results, writing of the manuscript, or the decision to submit the manuscript for publication.No financial grant or other assistance was gained related to this work.

## Ethics Statement

Ethical approval was not required for this study as it is a systematic review and meta‐analysis that exclusively utilizes previously published data. No human participants were directly involved, and no identifiable personal data were accessed.

## Conflicts of Interest

The authors declare no conflicts of interest.

## Guideline

This study follows the PRISMA guidelines for systematic reviews and meta‐analyses.

## Transparency Statement

The lead authors, Seyed Amir Ahmad Safavi‐Naini and Shabnam Shahrokh, affirm that this manuscript is an honest, accurate, and transparent account of the study being reported; that no important aspects of the study have been omitted; and that any discrepancies from the study as planned (and, if relevant, registered) have been explained.

## Supporting information

Supporting File

## Data Availability

All data generated or analyzed during this study are included in this published article. The analytic code and extracted dataset can be made available to facilitate replication of our results. The analytic methods (including R code used for meta‐analysis) and search strategies are available from the corresponding author upon reasonable request. No additional research materials beyond those described were used.
