# Supplementary material for: Diagnostic Efficacy and Clinical Aspects of Panoramic‐View Versus Forward‐View Capsule Endoscopy for Suspected Small Bowel Bleeding: A Systematic Review and Meta‐Analysis
Source: Health Sci Rep. 2026 May 3;9(5):e72095. doi: 10.1002/hsr2.72095 (PMC13136515; doi:10.1002/hsr2.72095)

**Supplementary Table 1**. Detailed search queries in each database.

| Database | Query |
| --- | --- |
| PubMed | ("gastrointestinal hemorrhage"[Title/Abstract] OR "gastrointestinal hemorrhages"[Title/Abstract] OR "obscure gastrointestinal bleeding"[Title/Abstract] OR "ogib"[Title/Abstract] OR "occult gi bleeding"[Title/Abstract] OR "overt gi bleeding"[Title/Abstract] OR "small bowel bleeding"[Title/Abstract] OR "obscure gi hemorrhage"[Title/Abstract] OR "occult gastrointestinal hemorrhage"[Title/Abstract] OR "overt gastrointestinal hemorrhage"[Title/Abstract] OR "occult gastrointestinal bleeding"[Title/Abstract] OR "overt gastrointestinal bleeding"[Title/Abstract] OR "obscure small bowel bleeding"[Title/Abstract] OR "unexplained gastrointestinal bleeding"[Title/Abstract] OR "gastrointestinal bleeding ogib"[Title/Abstract] OR "intermittent gastrointestinal bleeding"[Title/Abstract] OR "unexplained anemia"[Title/Abstract] OR "recurrent gastrointestinal bleeding"[Title/Abstract] OR "persistent gastrointestinal bleeding"[Title/Abstract] OR "difficult to diagnose lesions"[Title/Abstract] OR "suspected small bowel bleeding"[Title/Abstract] OR "chronic gastrointestinal bleeding"[Title/Abstract]) AND ("CapsoCam"[Title/Abstract] OR "Panoramic"[Title/Abstract] OR "Lateral View"[Title/Abstract] OR "four camera"[Title/Abstract] OR "side view"[Title/Abstract] OR "side-view"[Title/Abstract] OR "360"[Title/Abstract]) AND ("capsule endoscopy"[Title/Abstract] OR "wireless capsule endoscopy"[Title/Abstract] OR "video capsule endoscopy"[Title/Abstract] OR "capsule endoscopes"[Title/Abstract] OR "Capsule Endoscopes"[Title/Abstract] OR "Capsule endoscopy"[Title/Abstract] OR "Wireless endoscopy"[Title/Abstract] OR "Video Capsule Endoscope"[Title/Abstract]) AND ("pillcam"[Title/Abstract] OR "forward view"[Title/Abstract] OR "axial view"[Title/Abstract] OR "axial imaging"[Title/Abstract] OR "forward imaging"[Title/Abstract] OR "endocapsule"[Title/Abstract] OR "mirocam"[Title/Abstract] OR "omom"[Title/Abstract]) |
| Scopus | TITLE-ABS-KEY ( "gastrointestinal hemorrhage" OR "gastrointestinal hemorrhages" OR "obscure gastrointestinal bleeding" OR "ogib" OR "occult gi bleeding" OR "overt gi bleeding" OR "small bowel bleeding" OR "obscure gi hemorrhage" OR "occult gastrointestinal hemorrhage" OR "overt gastrointestinal hemorrhage" OR "occult gastrointestinal bleeding" OR "overt gastrointestinal bleeding" OR "obscure small bowel bleeding" OR "unexplained gastrointestinal bleeding" OR "gastrointestinal bleeding ogib" OR "intermittent gastrointestinal bleeding" OR "unexplained anemia" OR "recurrent gastrointestinal bleeding" OR "persistent gastrointestinal bleeding" OR "difficult to diagnose lesions" OR "suspected small bowel bleeding" OR "chronic gastrointestinal bleeding" ) AND TITLE-ABS-KEY ( "Capsocam" OR "Panoramic" OR "Lateral View" OR "four camera" OR "side view" OR "side-view" OR "360" ) AND TITLE-ABS-KEY ( "capsule endoscopy" OR "wireless capsule endoscopy" OR "video capsule endoscopy" OR "capsule endoscopes" OR "wireless endoscopy" OR "video capsule endoscope" ) AND TITLE-ABS-KEY ( "pillcam" OR "forward view" OR "axial view" OR "axial imaging" OR "forward imaging" OR "endocapsule" OR "mirocam" OR "omom" ) |
| Embase | ('gastrointestinal hemorrhage':ti,ab OR 'gastrointestinal hemorrhages':ti,ab OR 'obscure gastrointestinal bleeding':ti,ab OR 'ogib':ti,ab OR 'occult gi bleeding':ti,ab OR 'overt gi bleeding':ti,ab OR 'small bowel bleeding':ti,ab OR 'obscure gi hemorrhage':ti,ab OR 'occult gastrointestinal hemorrhage':ti,ab OR 'overt gastrointestinal hemorrhage':ti,ab OR 'occult gastrointestinal bleeding':ti,ab OR 'overt gastrointestinal bleeding':ti,ab OR 'obscure small bowel bleeding':ti,ab OR 'unexplained gastrointestinal bleeding':ti,ab OR 'gastrointestinal bleeding ogib':ti,ab OR 'intermittent gastrointestinal bleeding':ti,ab OR 'unexplained anemia':ti,ab OR 'recurrent gastrointestinal bleeding':ti,ab OR 'persistent gastrointestinal bleeding':ti,ab OR 'difficult to diagnose lesions':ti,ab OR 'suspected small bowel bleeding':ti,ab OR 'chronic gastrointestinal bleeding':ti,ab) AND ('capsocam':ti,ab OR 'panoramic':ti,ab OR 'lateral view':ti,ab OR 'four camera':ti,ab OR 'side view':ti,ab OR 'side-view':ti,ab OR '360':ti,ab) AND ('capsule endoscopy':ti,ab OR 'wireless capsule endoscopy':ti,ab OR 'video capsule endoscopy':ti,ab OR 'capsule endoscopes':ti,ab OR 'wireless endoscopy':ti,ab OR 'video capsule endoscope':ti,ab) AND ('pillcam':ti,ab OR 'forward view':ti,ab OR 'axial view':ti,ab OR 'axial imaging':ti,ab OR 'forward imaging':ti,ab OR 'endocapsule':ti,ab OR 'mirocam':ti,ab OR 'omom':ti,ab) |

**Supplementary Table 2.** The Joanna Briggs Institute checklist for quality assessment.

| **Question** | **Answers and Scores** | | | |  |
| --- | --- | --- | --- | --- | --- |
| 1. Was a consecutive or random sample of patients enrolled? | Yes = 1 | No = 0 | Unknown= 0 | Not applicable = 0 | |
| 2. Was a case control design avoided? | Yes = 1 | No = 0 | Unknown= 0 | Not applicable = 0 | |
| 3. Did the study avoid inappropriate exclusions? | Yes = 1 | No = 0 | Unknown= 0 | Not applicable = 0 | |
| 4. Were the index test results interpreted without knowledge of the results of the reference standard? | Yes = 1 | No = 0 | Unknown= 0 | Not applicable = 0 | |
| 5. If a threshold was used, was it pre-specified? | Yes = 1 | No = 0 | Unknown= 0 | Not applicable = 0 | |
| 6. Is the reference standard likely to correctly classify the target condition? | Yes = 1 | No = 0 | Unknown= 0 | Not applicable = 0 | |
| 7. Were the reference standard results interpreted without knowledge of the results of the index test? | Yes = 1 | No = 0 | Unknown= 0 | Not applicable = 0 | |
| 8. Was there an appropriate interval between index test and reference standard? | Yes = 1 | No = 0 | Unknown= 0 | Not applicable = 0 | |
| 9. Did all patients receive the same reference standard? | Yes = 1 | No = 0 | Unknown= 0 | Not applicable = 0 | |
| 10. Were all patients included in the analysis? | Yes = 1 | No = 0 | Unknown= 0 | Not applicable = 0 | |

**Supplementary Table 3.** Results of the subgroup meta-analysis for different panoramic capsule types. RD, Risk Difference; SMD, Standardized Mean Difference; N, Number; CI, Confidence Interval.

| RD (Risk Difference) — subgroup results | | |  |  |  |  |
| --- | --- | --- | --- | --- | --- | --- |
| Variable | Subgroup | N.studies | Effect.size | CI.low | CI.high | P.value |
| Diagnostic_Yield | Capsocam | 1 |  |  |  |  |
| Diagnostic_Yield | CapsoCam PLUS | 2 | -0.04 | -0.21 | 0.14 | P = 0.22 |
| Diagnostic_Yield | CapsoCam SV-1 | 2 | 0.02 | -0.4 | 0.45 | P = 0.63 |
| Completion | CapsoCam PLUS | 2 | 0.16 | -2.19 | 2.51 | P = 0.55 |
| Completion | CapsoCam SV-1 | 2 | -0.08 | -0.73 | 0.57 | P = 0.37 |
| Retention | CapsoCam PLUS | 2 | 0 | 0 | 0 |  |
| Retention | CapsoCam SV-1 | 2 | 0.01 | -0.22 | 0.25 | P = 0.60 |
| Technical_Fault | CapsoCam PLUS | 2 | -0.17 | -2.54 | 2.2 | P = 0.53 |
| Technical_Fault | CapsoCam SV-1 | 2 | 0.02 | -0.21 | 0.25 | P = 0.42 |
| Incomplete_Transit | CapsoCam PLUS | 2 | -0.14 | -1.66 | 1.39 | P = 0.46 |
| Incomplete_Transit | CapsoCam SV-1 | 2 | 0 | -0.06 | 0.05 | P = 0.84 |
| Insufficience_Preparation | CapsoCam PLUS | 2 | 0 | 0 | 0 |  |
| Insufficience_Preparation | CapsoCam SV-1 | 2 | 0 | -0.14 | 0.13 | P = 0.72 |
| SMD (Standardized Mean Difference) — subgroup results | | | | |  |  |
| Variable | Subgroup | N.studies | Effect.size | CI.low | CI.high | P.value |
| Small_Bowel_Transit_Time | CapsoCam PLUS | 2 | -0.11 | -2 | 1.78 | P = 0.60 |
| Small_Bowel_Transit_Time | CapsoCam SV-1 | 2 | 0.34 | -4.72 | 5.4 | P = 0.55 |
| Gastric_Transit_Time | CapsoCam PLUS | 2 | -0.03 | -0.21 | 0.15 | P = 0.27 |
| Gastric_Transit_Time | CapsoCam SV-1 | 1 |  |  |  |  |
| Reading_Time | CapsoCam PLUS | 2 | 0.65 | -2.06 | 3.36 | P = 0.20 |
| Reading_Time | CapsoCam SV-1 | 2 | 1.36 | -4.33 | 7.05 | P = 0.20 |

**Supplementary Table 4.** Results of the subgroup meta-analysis for different study designs. RD, Risk Difference; SMD, Standardized Mean Difference; N, Number; CI, Confidence Interval; RCT, Randomized Controlled Trial.

| RD (Risk Difference) — subgroup results | | |  |  |  |  |
| --- | --- | --- | --- | --- | --- | --- |
| Variable | Subgroup | N.studies | Effect.size | CI.low | CI.high | P.value |
| Diagnostic_Yield | RCT | 3 | 0.01 | -0.11 | 0.14 | P = 0.69 |
| Diagnostic_Yield | Retrospective | 2 | -0.01 | -0.33 | 0.32 | P = 0.85 |
| Completion | RCT | 3 | 0.04 | -0.57 | 0.65 | P = 0.79 |
| Completion | Retrospective | 1 |  |  |  |  |
| Retention | RCT | 3 | 0.01 | -0.04 | 0.07 | P = 0.43 |
| Retention | Retrospective | 1 |  |  |  |  |
| Technical_Fault | RCT | 3 | -0.08 | -0.63 | 0.48 | P = 0.62 |
| Technical_Fault | Retrospective | 1 |  |  |  |  |
| Incomplete_Transit | RCT | 3 | 0 | -0.01 | 0.01 | P = 0.75 |
| Incomplete_Transit | Retrospective | 1 |  |  |  |  |
| Insufficience_Preparation | RCT | 3 | 0 | -0.04 | 0.03 | P = 0.58 |
| Insufficience_Preparation | Retrospective | 1 |  |  |  |  |
| SMD (Standardized Mean Difference) — subgroup results | | | | |  |  |
| Variable | Subgroup | N.studies | Effect.size | CI.low | CI.high | P.value |
| Small_Bowel_Transit_Time | RCT | 3 | 0.16 | -1.21 | 1.53 | P = 0.66 |
| Small_Bowel_Transit_Time | Retrospective | 1 |  |  |  |  |
| Gastric_Transit_Time | RCT | 2 | -0.22 | -1.3 | 0.87 | P = 0.24 |
| Gastric_Transit_Time | Retrospective | 1 |  |  |  |  |
| Reading_Time | RCT | 3 | 1.06 | -0.74 | 2.87 | P = 0.13 |
| Reading_Time | Retrospective | 1 |  |  |  |  |

**Supplementary Figure 1.** Results of Sensitivity Analysis on Diagnostic Yield to Evaluate the Robustness of the Meta-Analysis. CI: Confidence Interval.


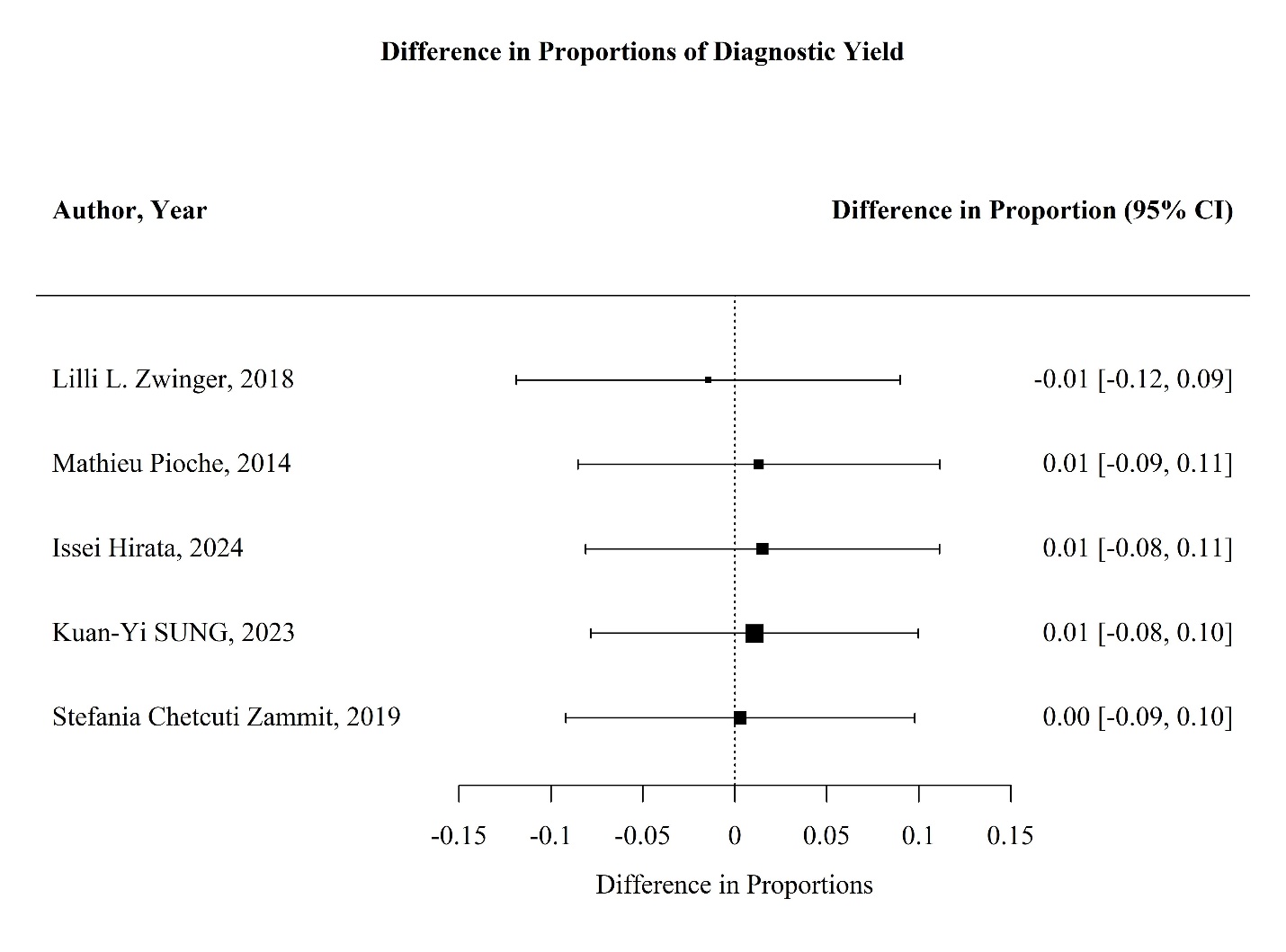


**Supplementary Figure 2**. **a:** Forest plot of gastric transit time of capsules. **b:** Funnel plot of gastric transit time of capsules. **c:** Galbraith plot of gastric transit time of capsules. CE, capsule endoscopy; N, number; SD, standard deviation; CI, confidence interval.


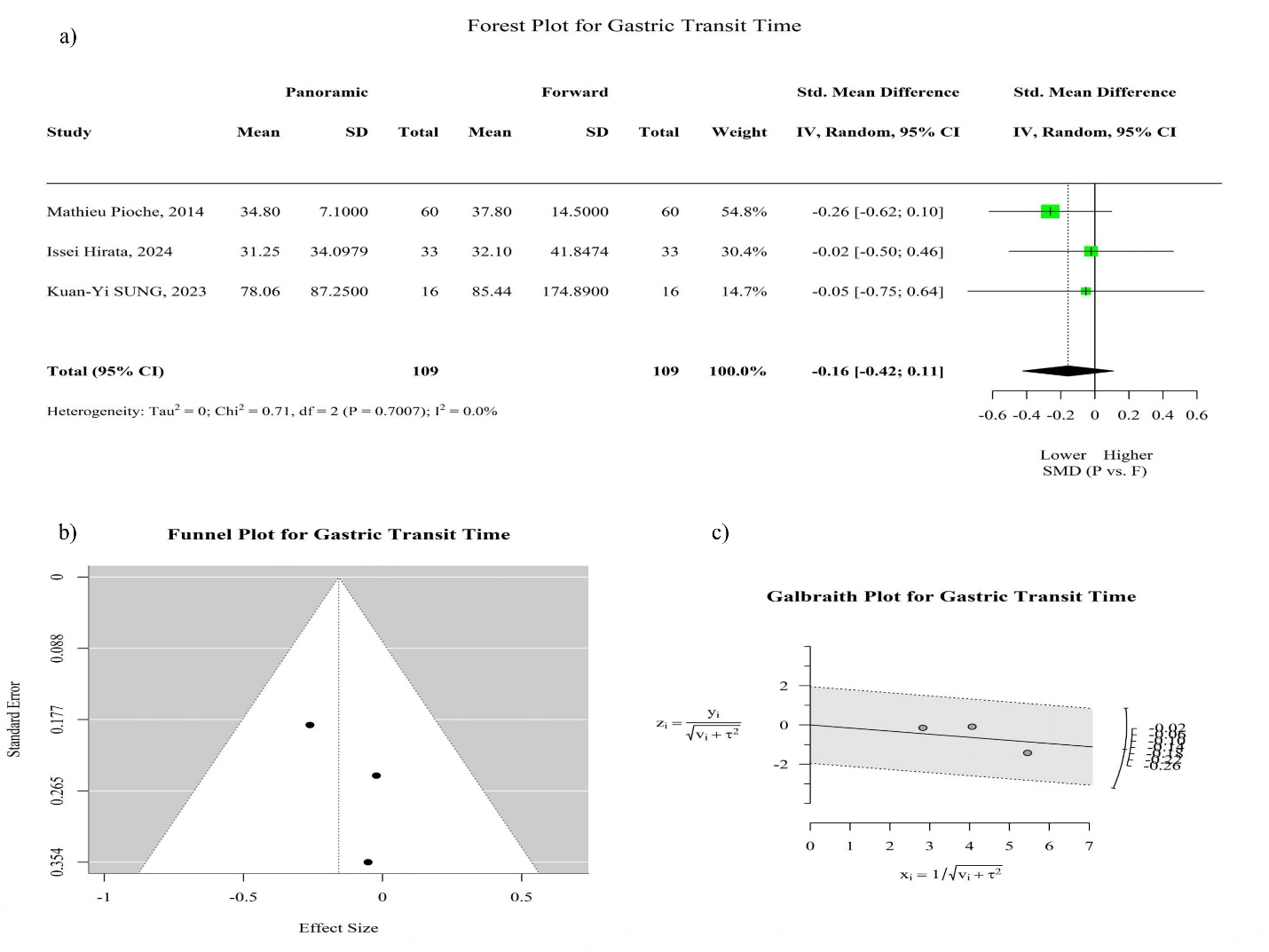


**Supplementary Figure 3.** Results of Sensitivity Analysis on Small Bowel Transit Time to Evaluate the Robustness of the Meta-Analysis. CI: Confidence Interval.


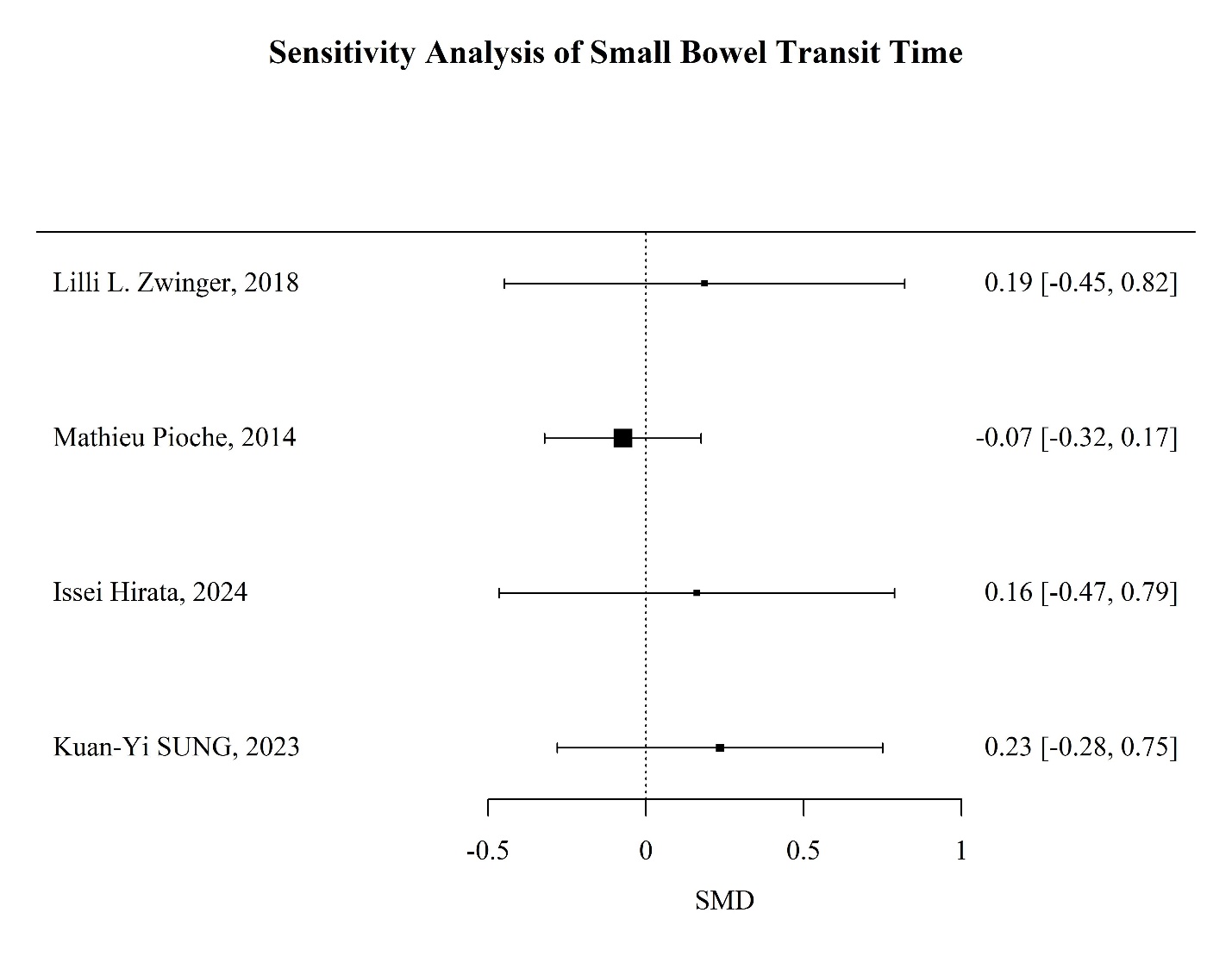


**Supplementary Figure 4.** Results of Sensitivity Analysis on Gastric Transit Time to Evaluate the Robustness of the Meta-Analysis. CI: Confidence Interval.


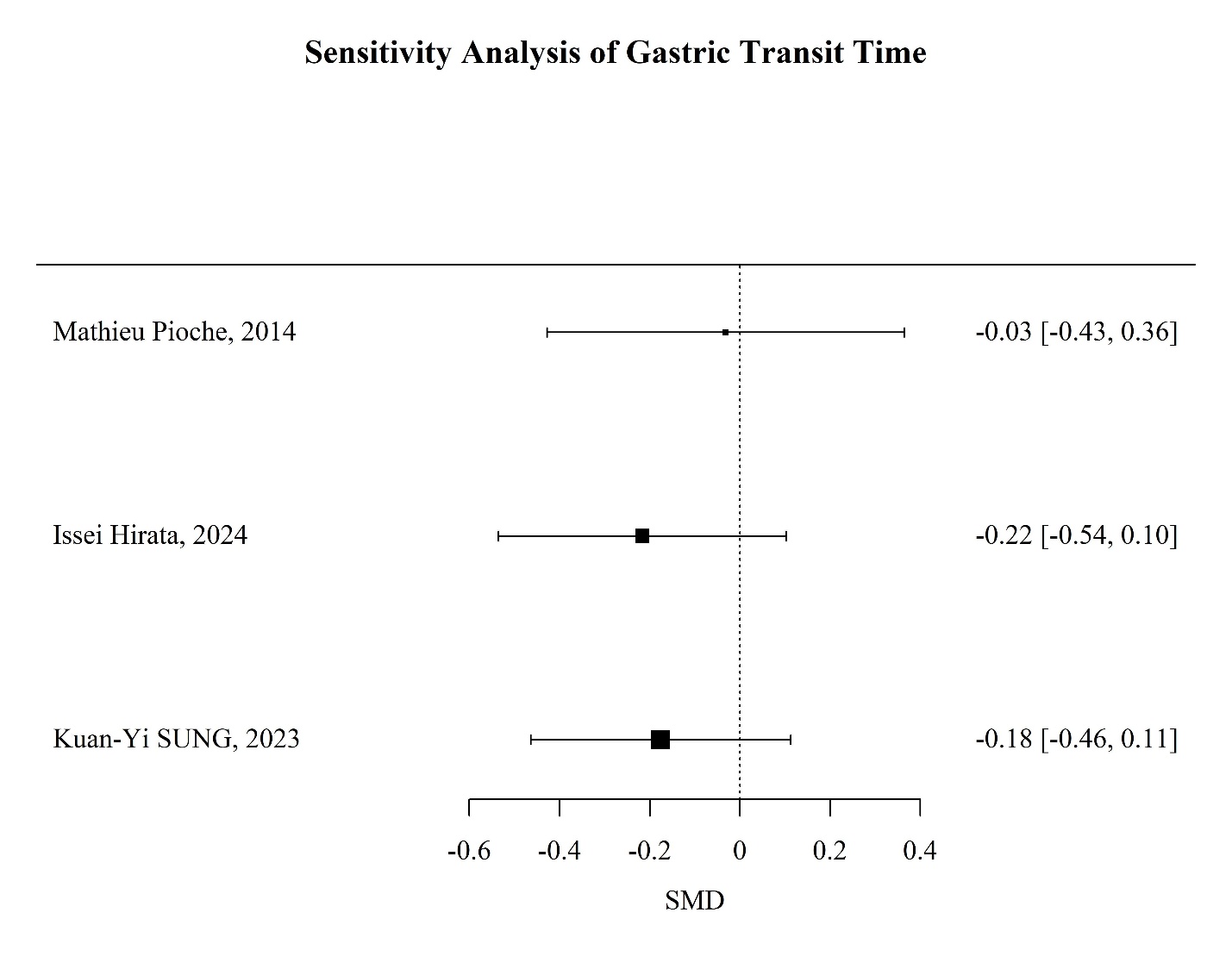


**Supplementary Figure 5.** Results of Sensitivity Analysis on Reading Time to Evaluate the Robustness of the Meta-Analysis. CI: Confidence Interval.


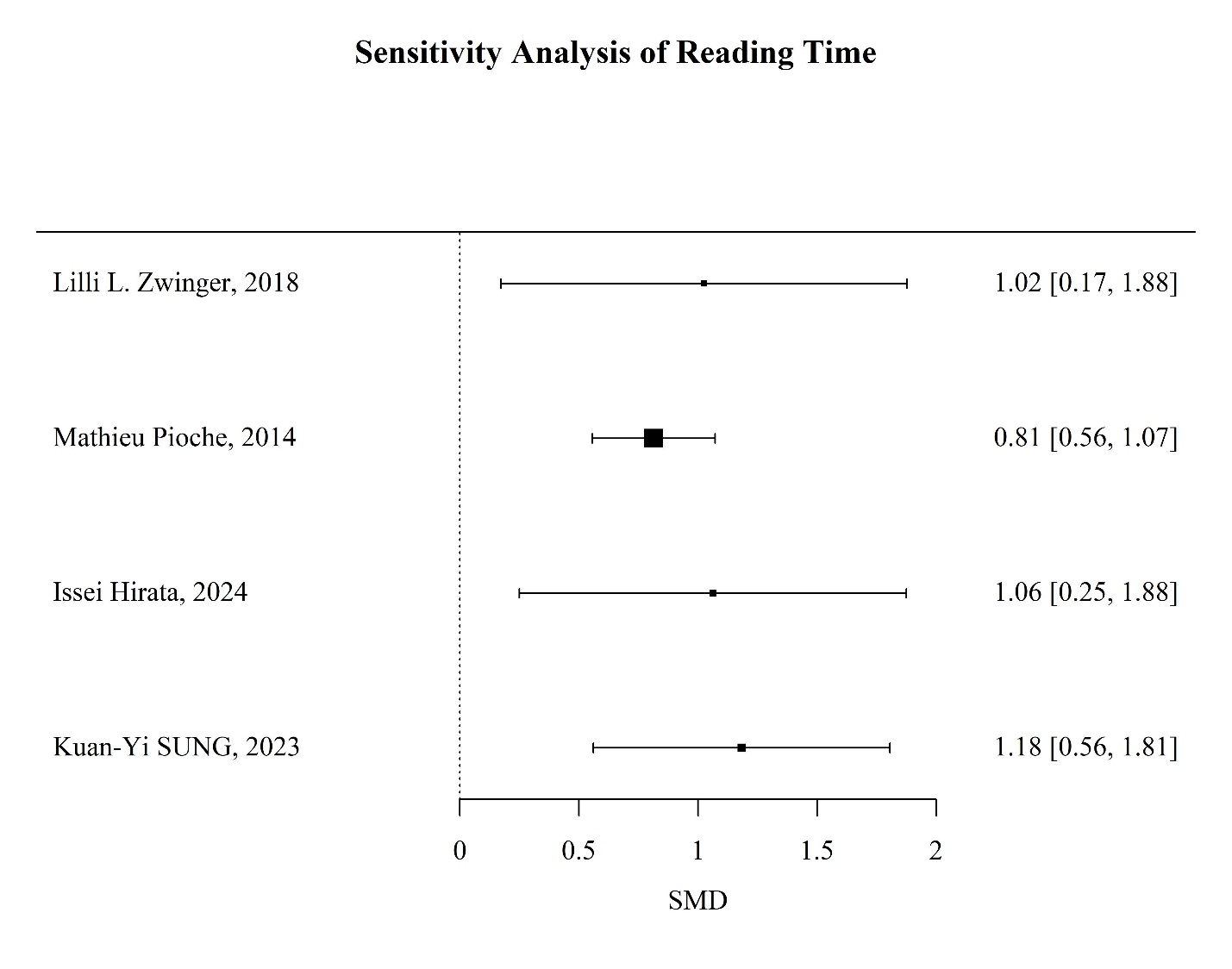


**Supplementary Figure 6.** Results of Sensitivity Analysis on Completion to Evaluate the Robustness of the Meta-Analysis. CI: Confidence Interval.


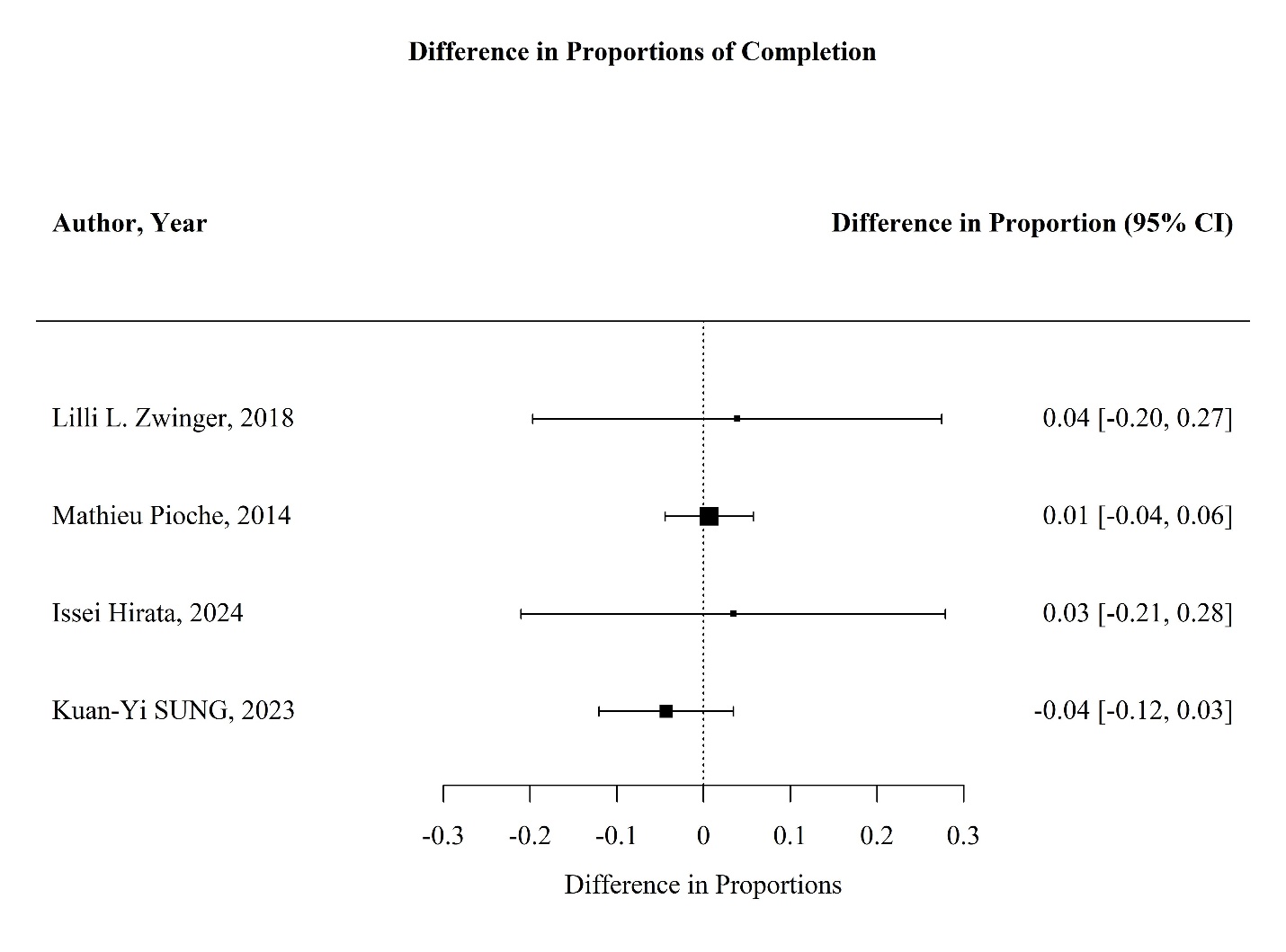


**Supplementary Figure 7.** Results of Sensitivity Analysis on Incomplete Transit to Evaluate the Robustness of the Meta-Analysis. CI: Confidence Interval.


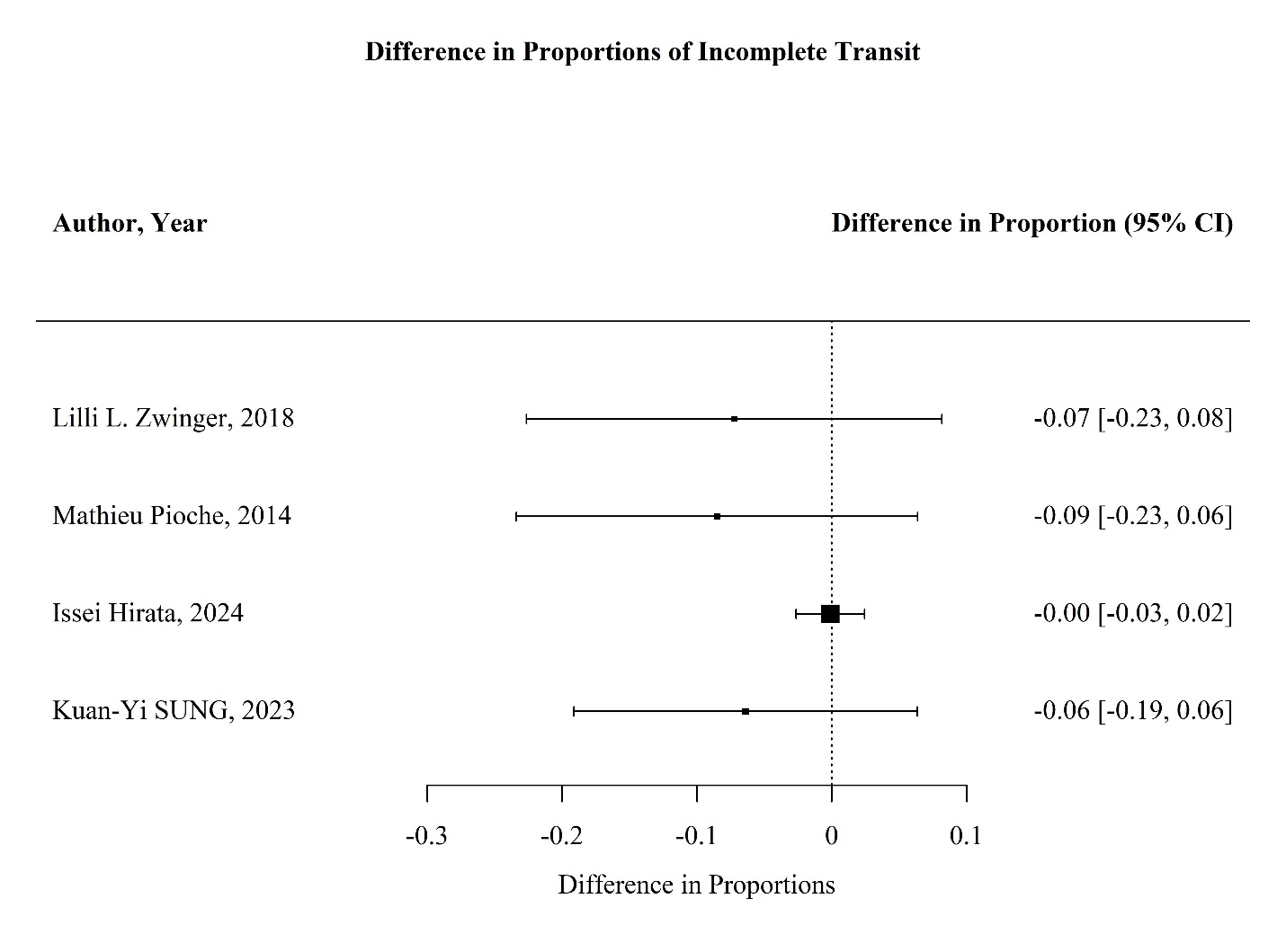


**Supplementary Figure 8.** Results of Sensitivity Analysis on Retention to Evaluate the Robustness of the Meta-Analysis. CI: Confidence Interval.

**
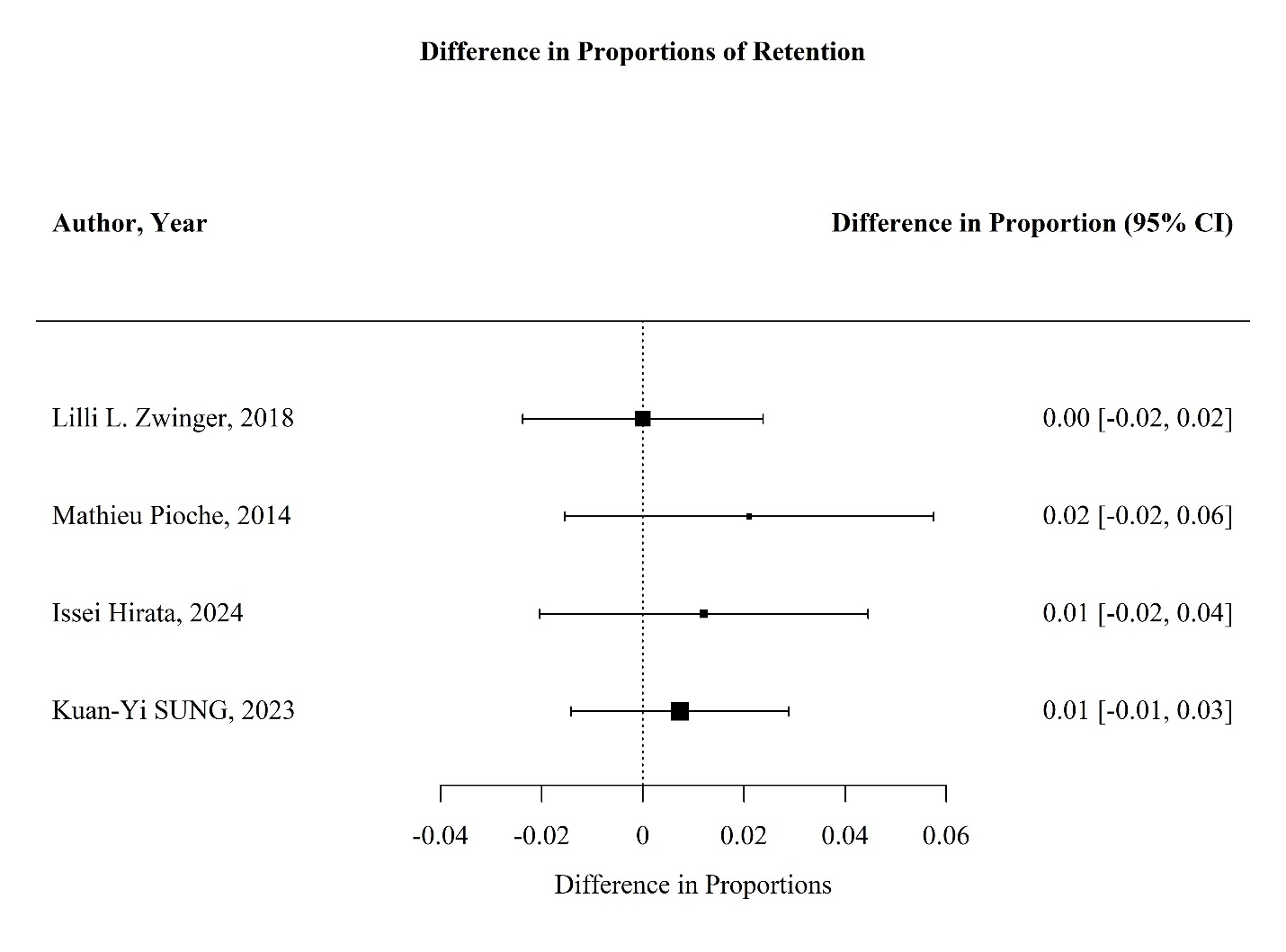
**

**Supplementary Figure 9.** Results of Sensitivity Analysis on Technical Fault to Evaluate the Robustness of the Meta-Analysis. CI: Confidence Interval.


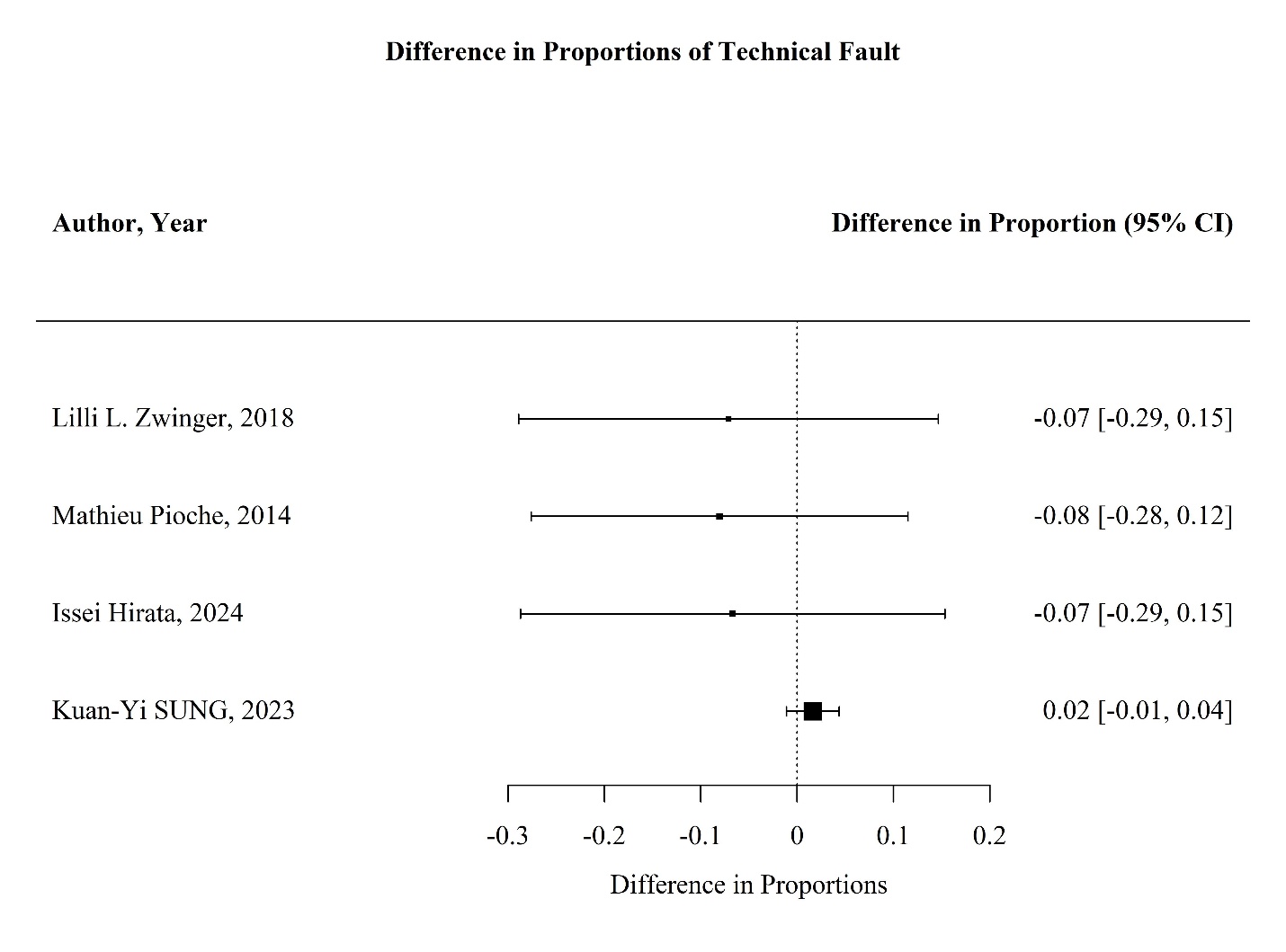


**Supplementary Figure 10.** **a:** Forest plot of technical fault rate of capsules. **b:** Funnel plot of technical fault rate of capsules. **c:** Galbraith plot of technical fault rate of capsules. SD, standard deviation; Std, standard; SMD, standardized mean difference; CI, confidence interval.


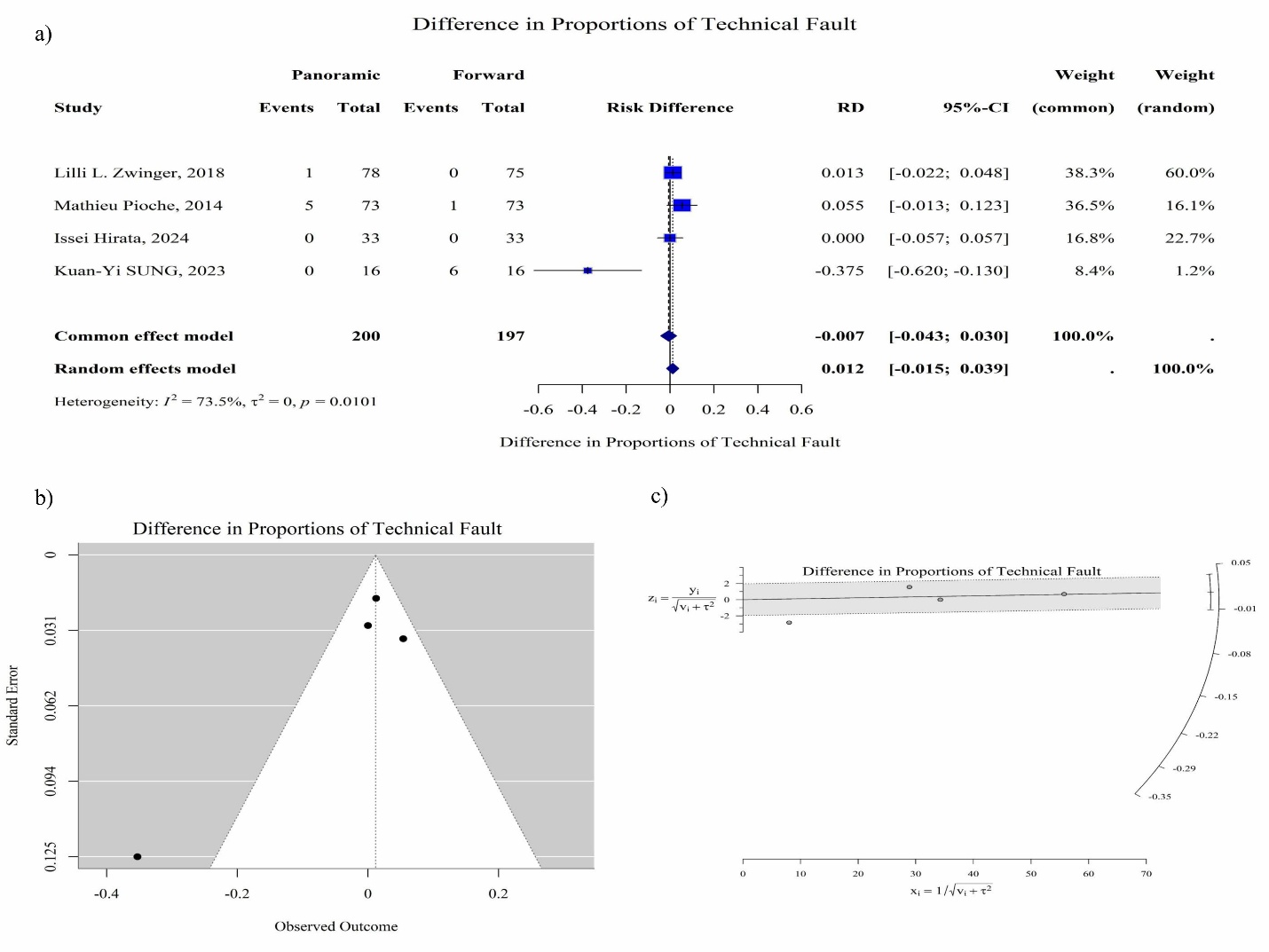


**Supplementary Figure 11.** **a:** Forest plot of retention rate of capsules. **b:** Funnel plot of retention rate of capsules. **c:** Galbraith plot of retention rate of capsules. RD, risk difference; CI, confidence interval.


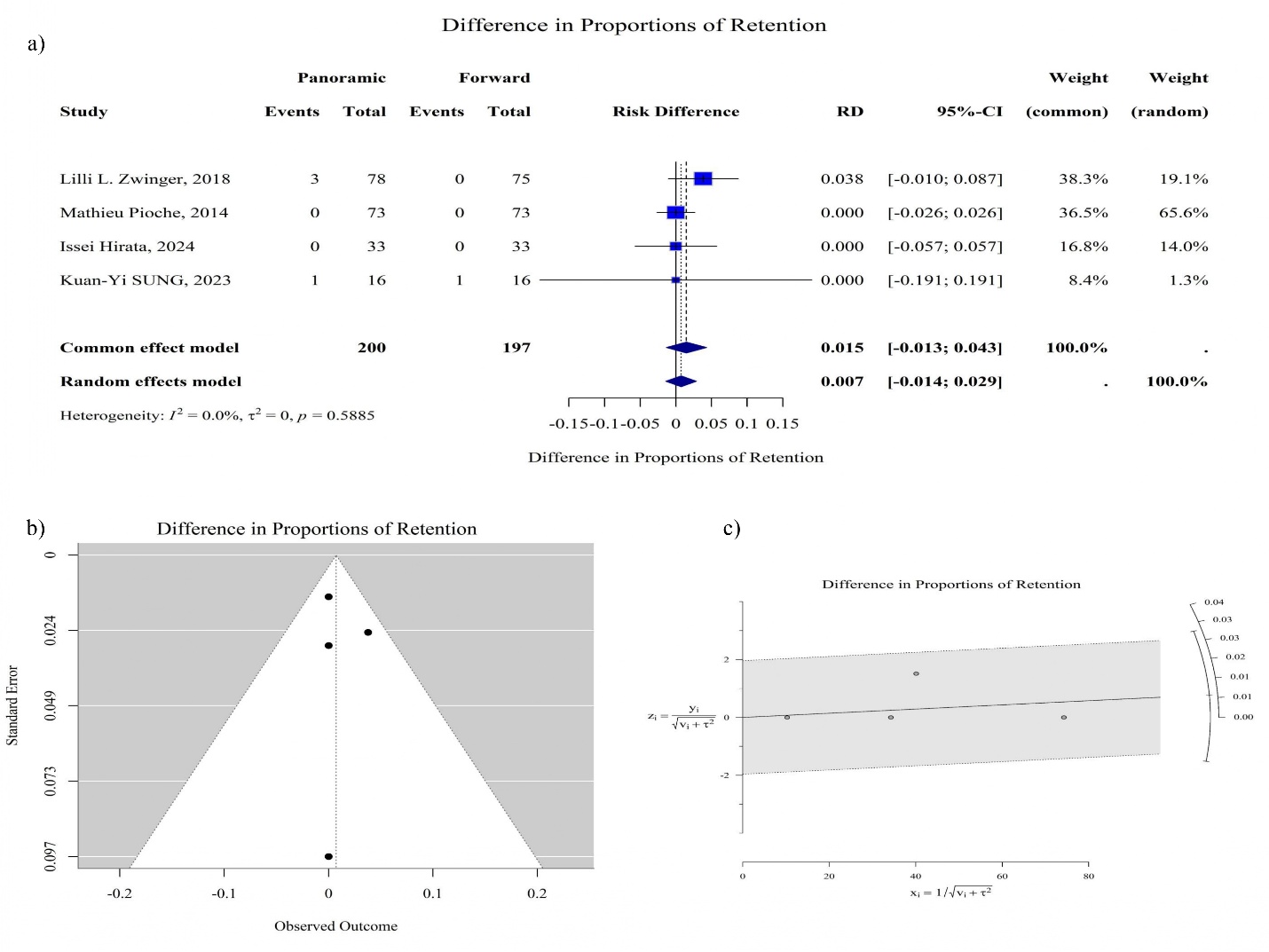


**Supplementary Figure 12.** **a:** Forest plot of incomplete transit rate of capsules. **b:** Funnel plot of incomplete transit rate of capsules. **c:** Galbraith plot of incomplete transit rate of capsules. RD, risk difference; CI, confidence interval.


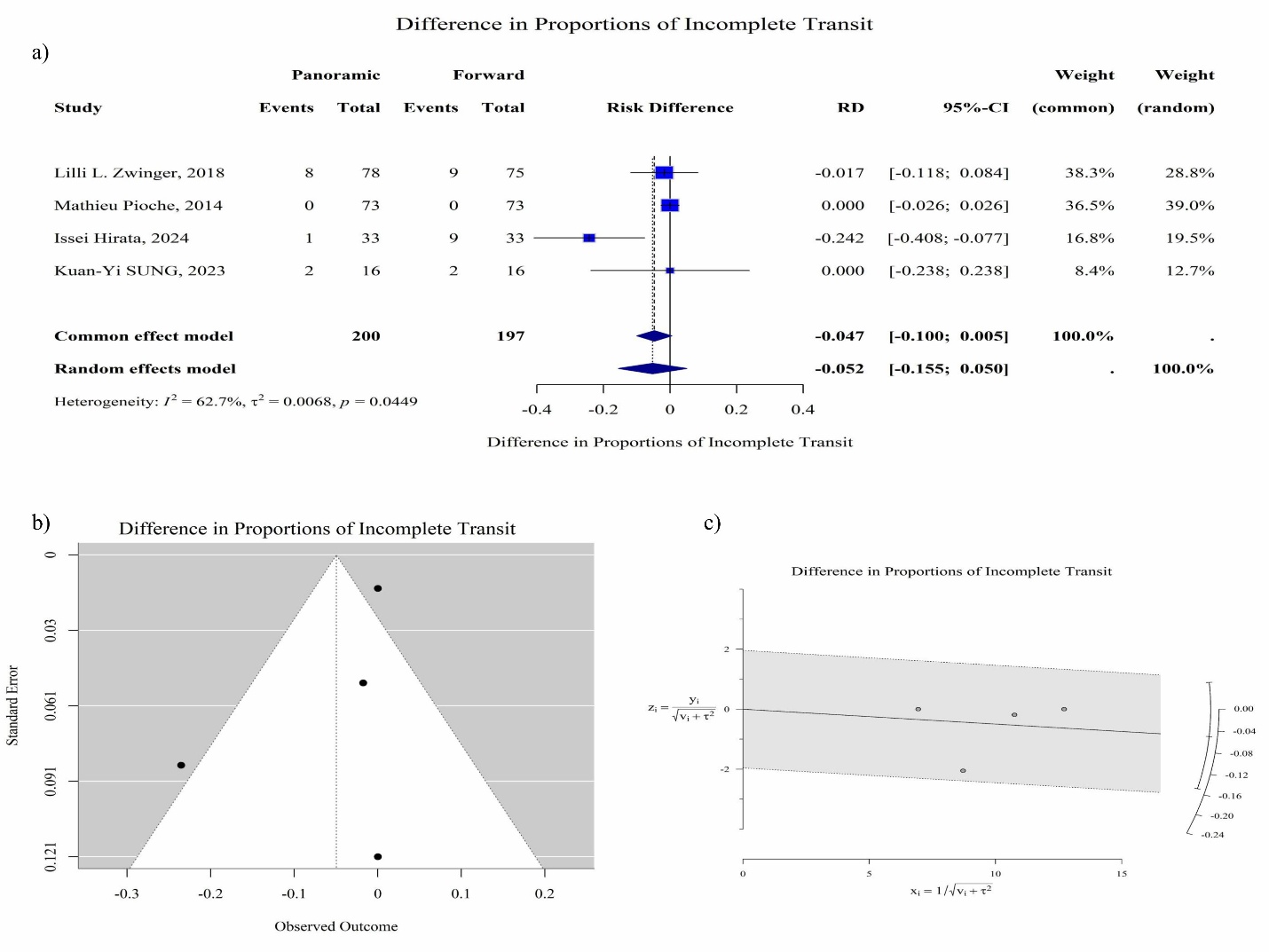


**Supplementary Figure 13.** Forest plot of subgroup analysis by CapsoCam generation for the diagnostic yield of capsule endoscopy. RD, risk difference; MH, Mantel-Haenszel

CI, confidence interval.


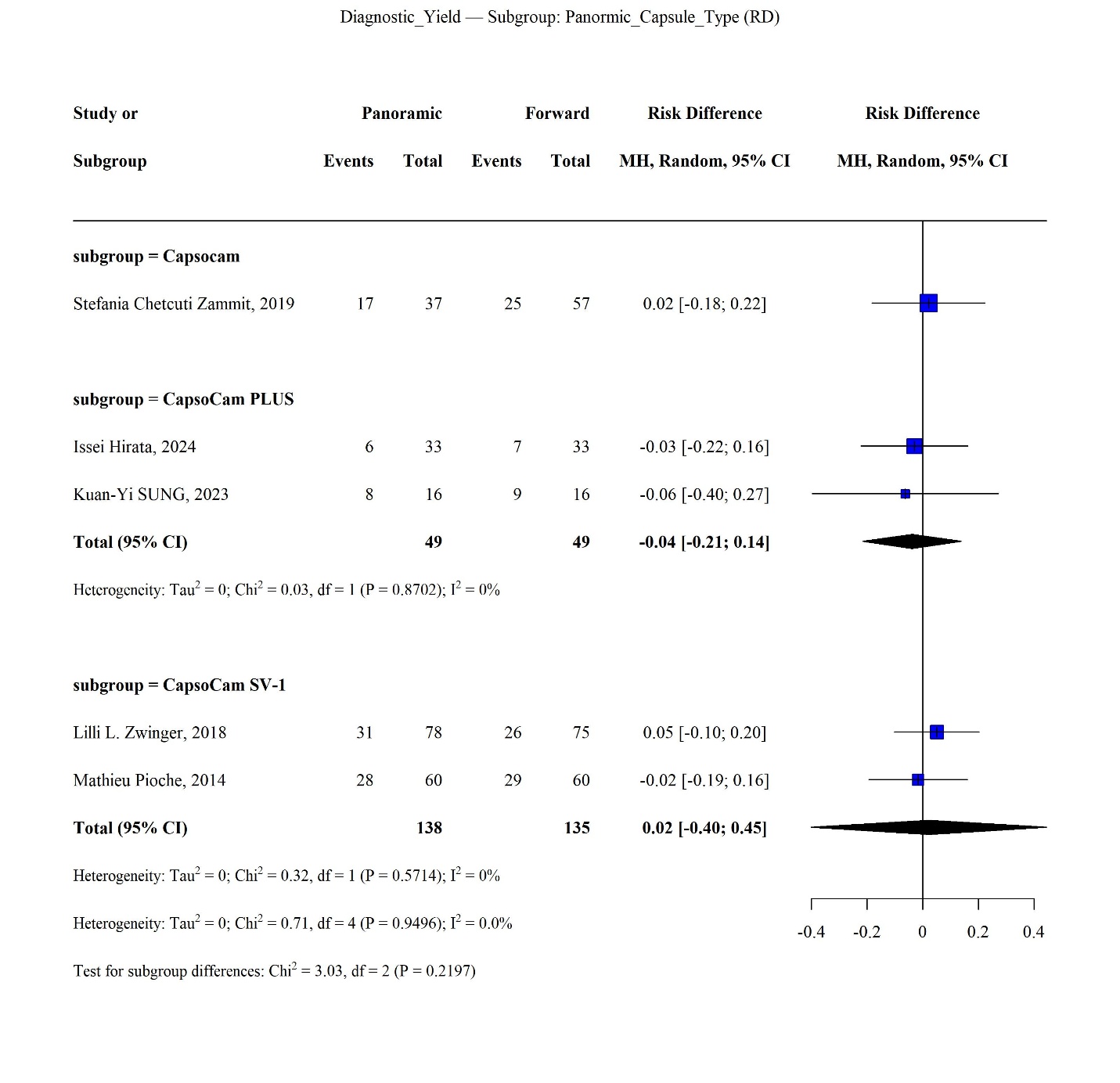


**Supplementary Figure 14.** Forest plot of subgroup analysis by CapsoCam generation for the small bowel transit time of capsule endoscopy. SMD, Standardized Mean Difference; SD, Standard Deviation; CI, confidence interval; IV, Inverse variance.


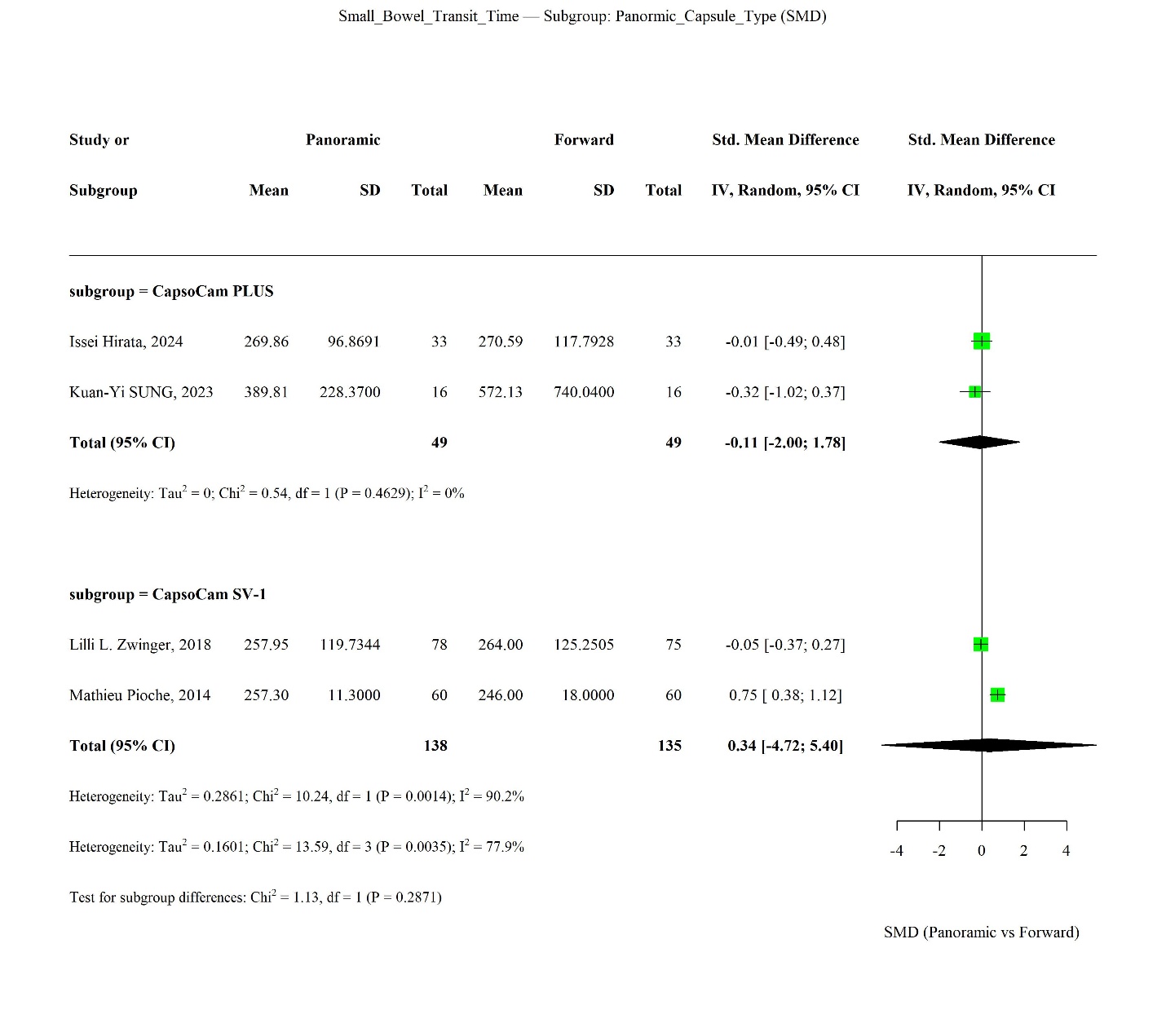


**Supplementary Figure 15.** Forest plot of subgroup analysis by CapsoCam generation for the gastric transit time of capsule endoscopy. SMD, Standardized Mean Difference; SD, Standard Deviation; CI, confidence interval; IV, Inverse variance.


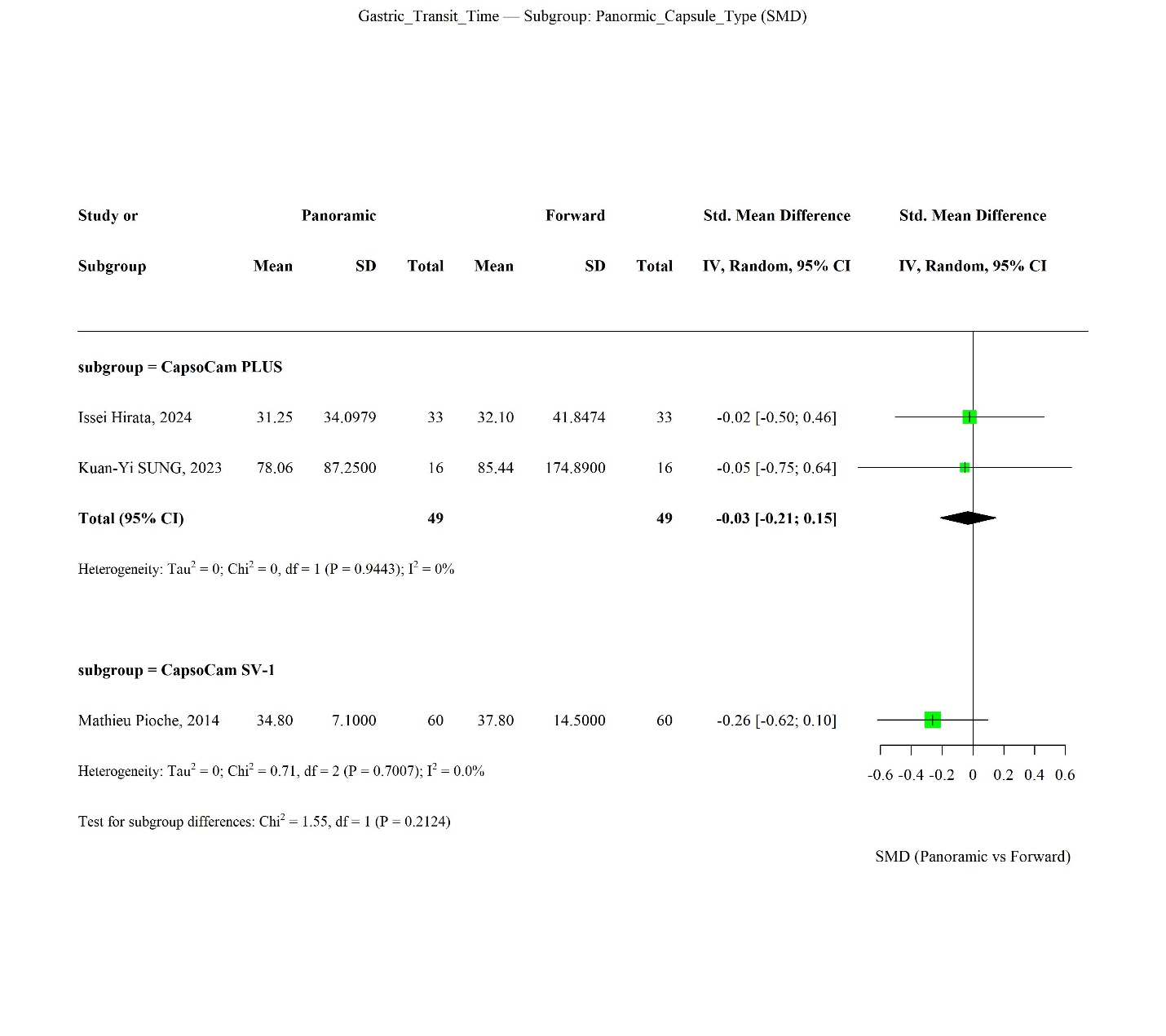


**Supplementary Figure 16.** Forest plot of subgroup analysis by CapsoCam generation for the reading time of capsule endoscopy. SMD, Standardized Mean Difference; SD, Standard Deviation; CI, confidence interval; IV, Inverse variance.


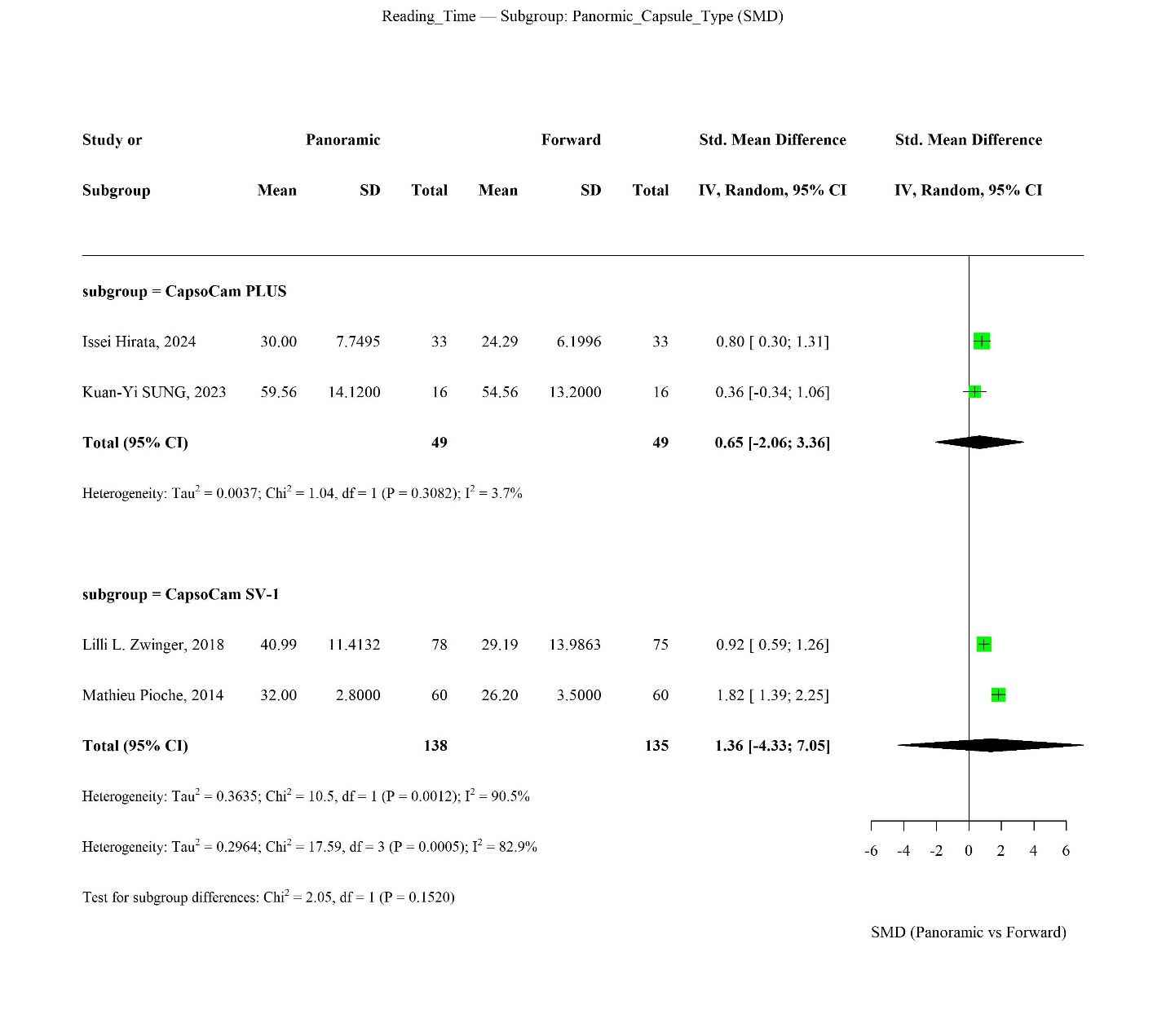


**Supplementary Figure 17.** Forest plot of subgroup analysis by CapsoCam generation for the completion of capsule endoscopy. RD, risk difference; MH, Mantel-Haenszel

CI, confidence interval.


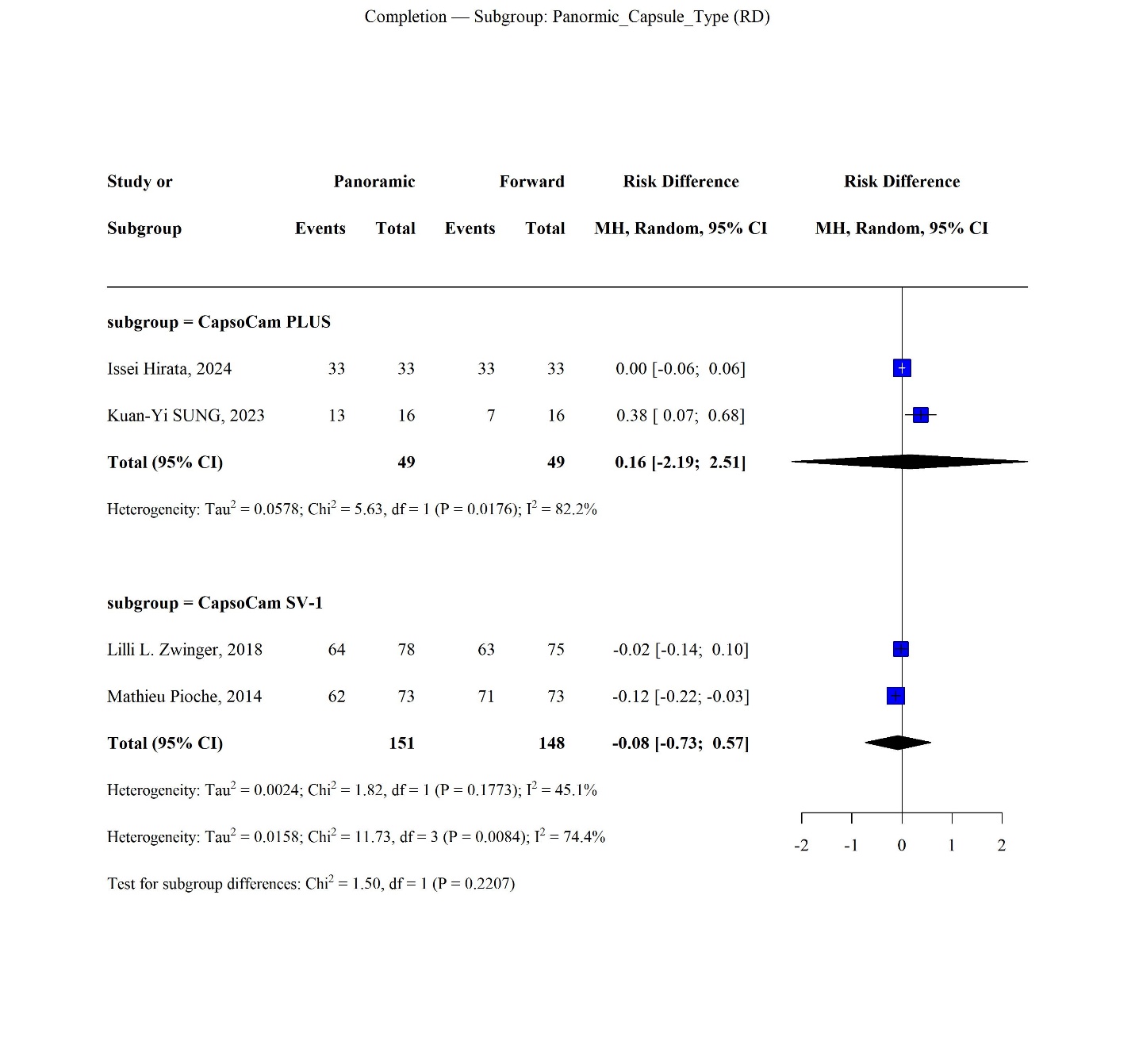


**Supplementary Figure 18.** Forest plot of subgroup analysis by CapsoCam generation for the technical fault of capsule endoscopy. RD, risk difference; MH, Mantel-Haenszel

CI, confidence interval.


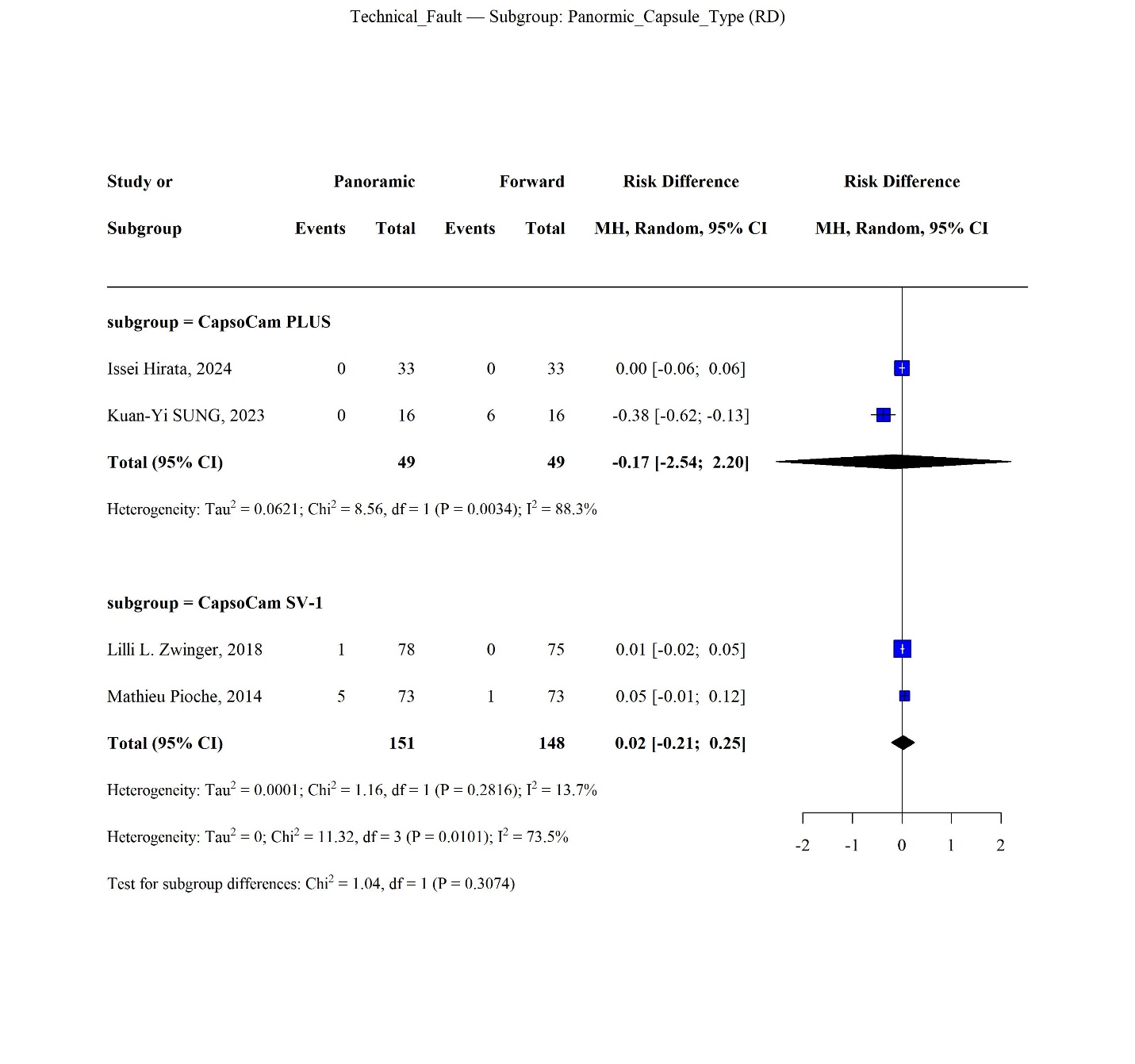


**Supplementary Figure 19.** Forest plot of subgroup analysis by CapsoCam generation for the retention of capsule endoscopy. RD, risk difference; MH, Mantel-Haenszel

CI, confidence interval.


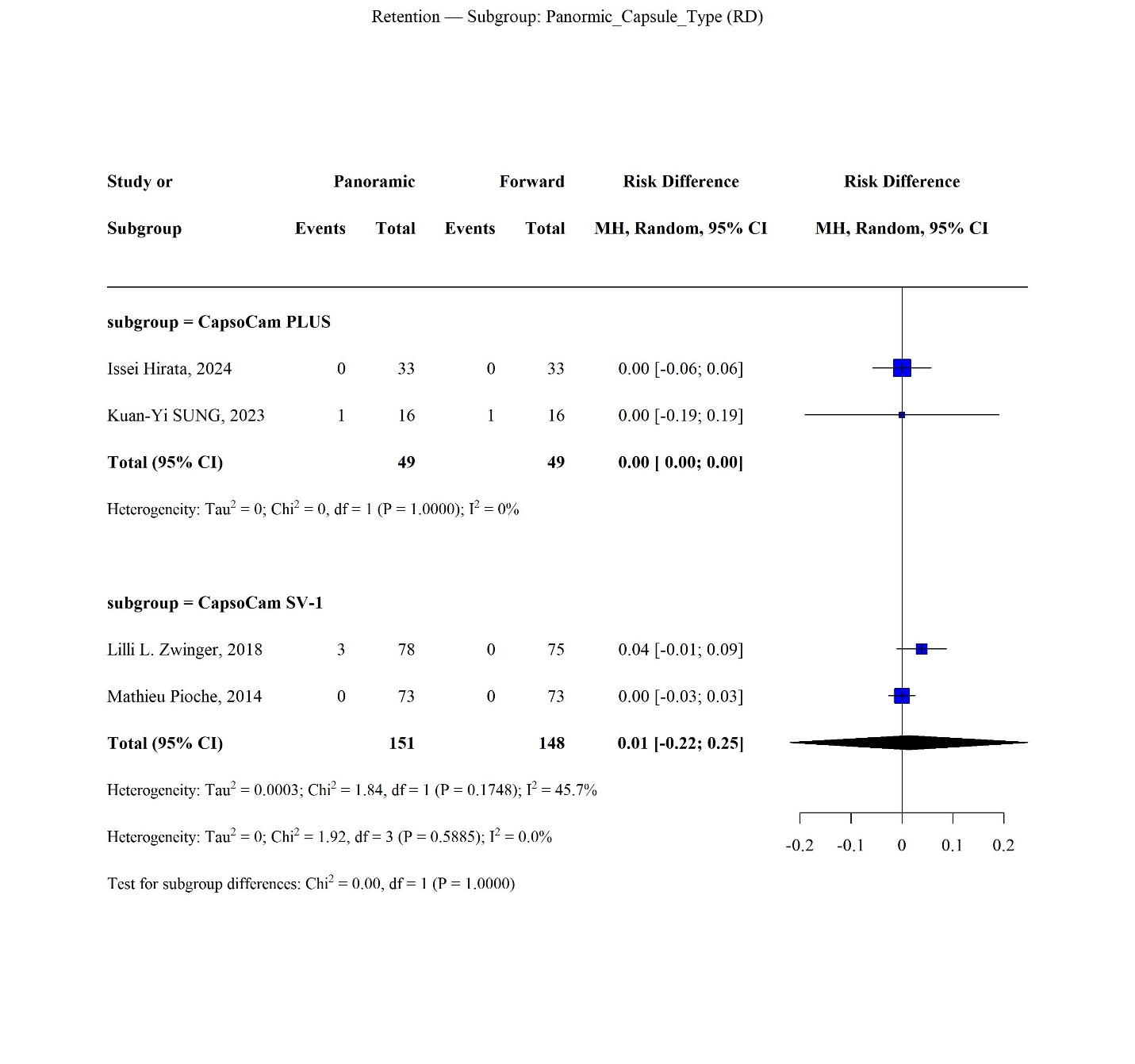


**Supplementary Figure 20.** Forest plot of subgroup analysis by CapsoCam generation for the incomplete transit of capsule endoscopy. RD, risk difference; MH, Mantel-Haenszel

CI, confidence interval.


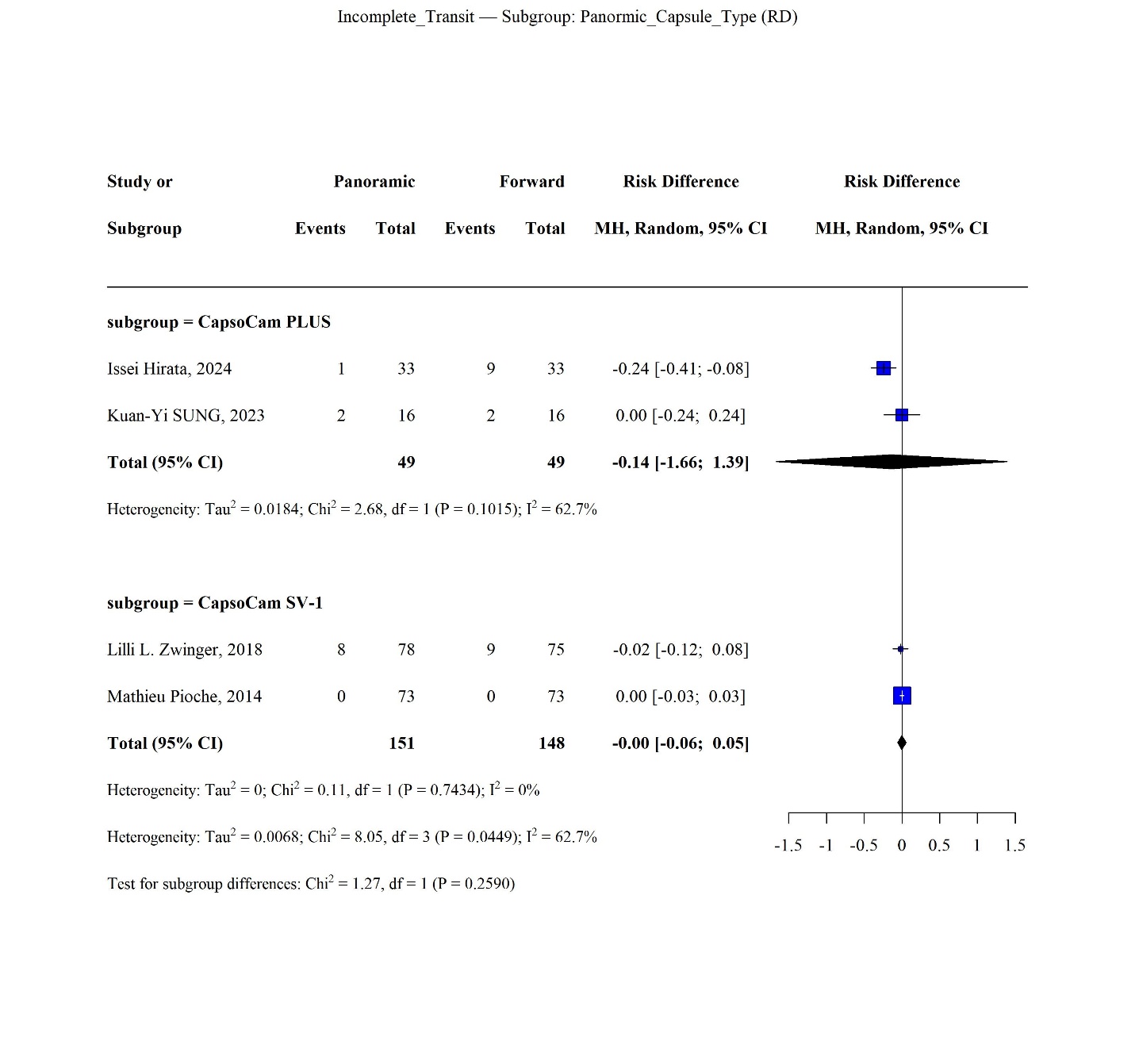


**Supplementary Figure 21.** Forest plot of subgroup analysis by CapsoCam generation for the insufficient preparation of capsule endoscopy. RD, risk difference; MH, Mantel-Haenszel

CI, confidence interval.


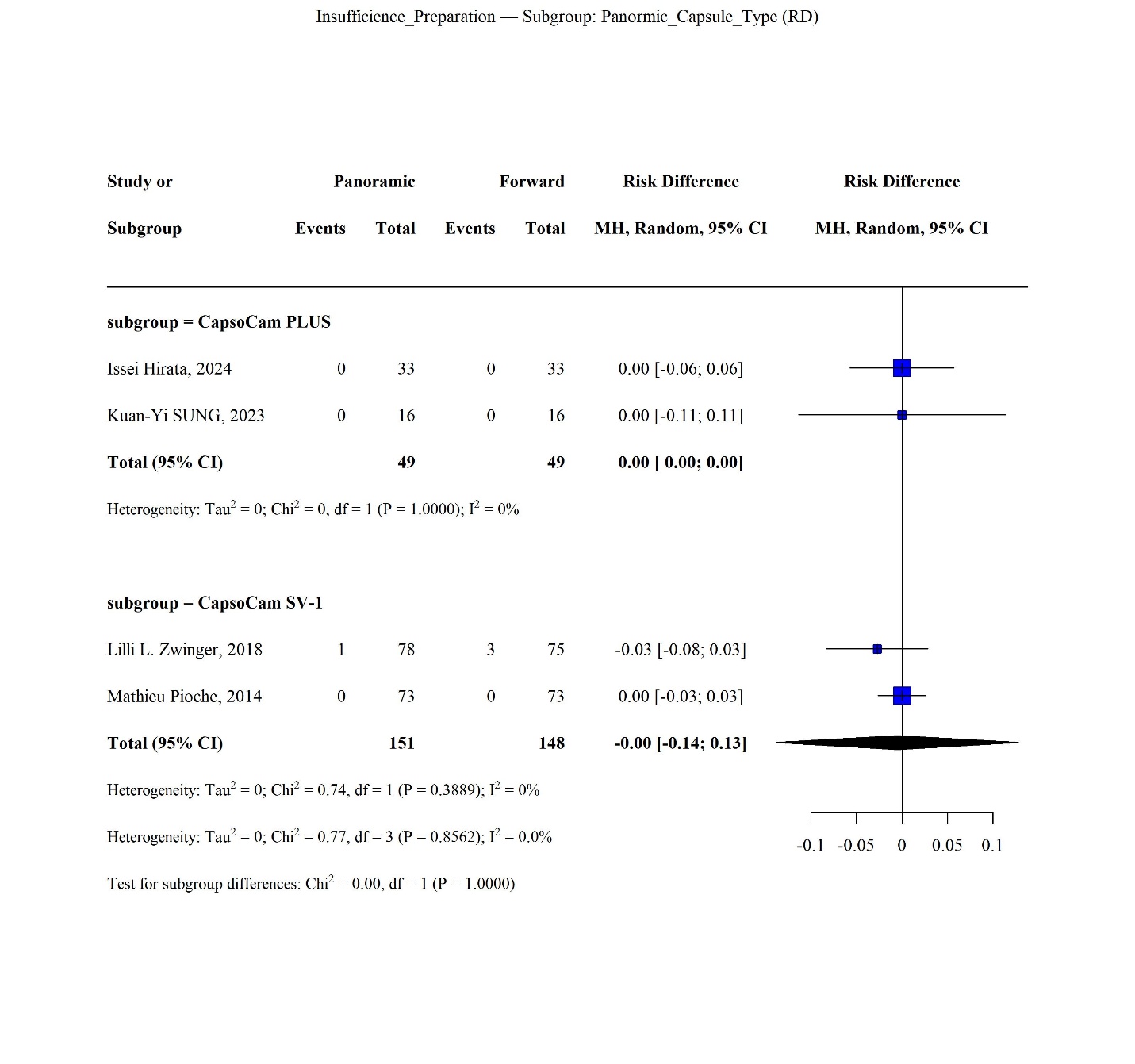


**Supplementary Figure 22.** Forest plot of subgroup analysis by study design for the diagnostic yield of capsule endoscopy. RD, risk difference; MH, Mantel-Haenszel

CI, confidence interval.


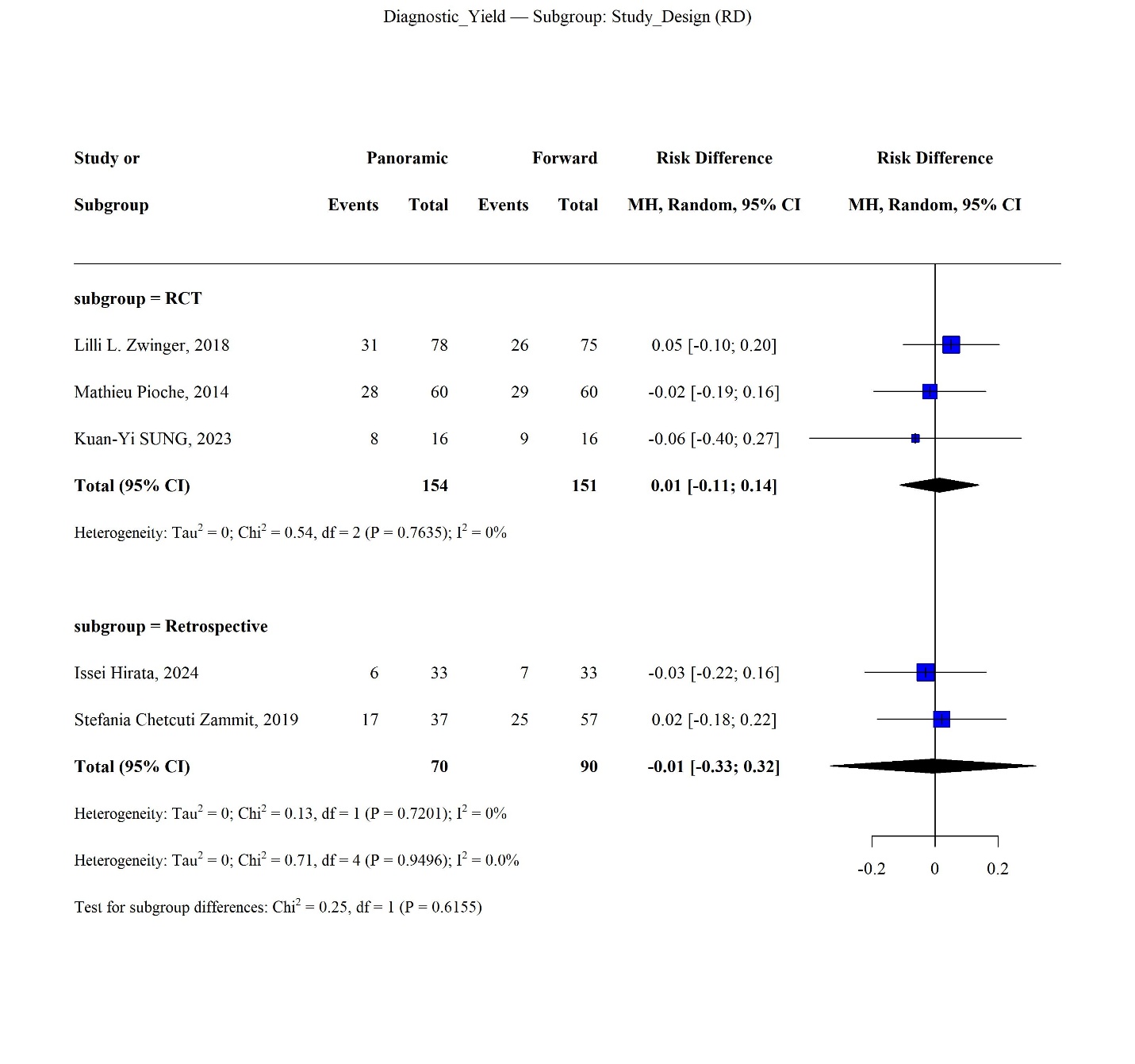


**Supplementary Figure 23.** Forest plot of subgroup analysis by study design for the small bowel transit time of capsule endoscopy. SMD, Standardized Mean Difference; SD, Standard Deviation; CI, confidence interval; IV, Inverse variance.


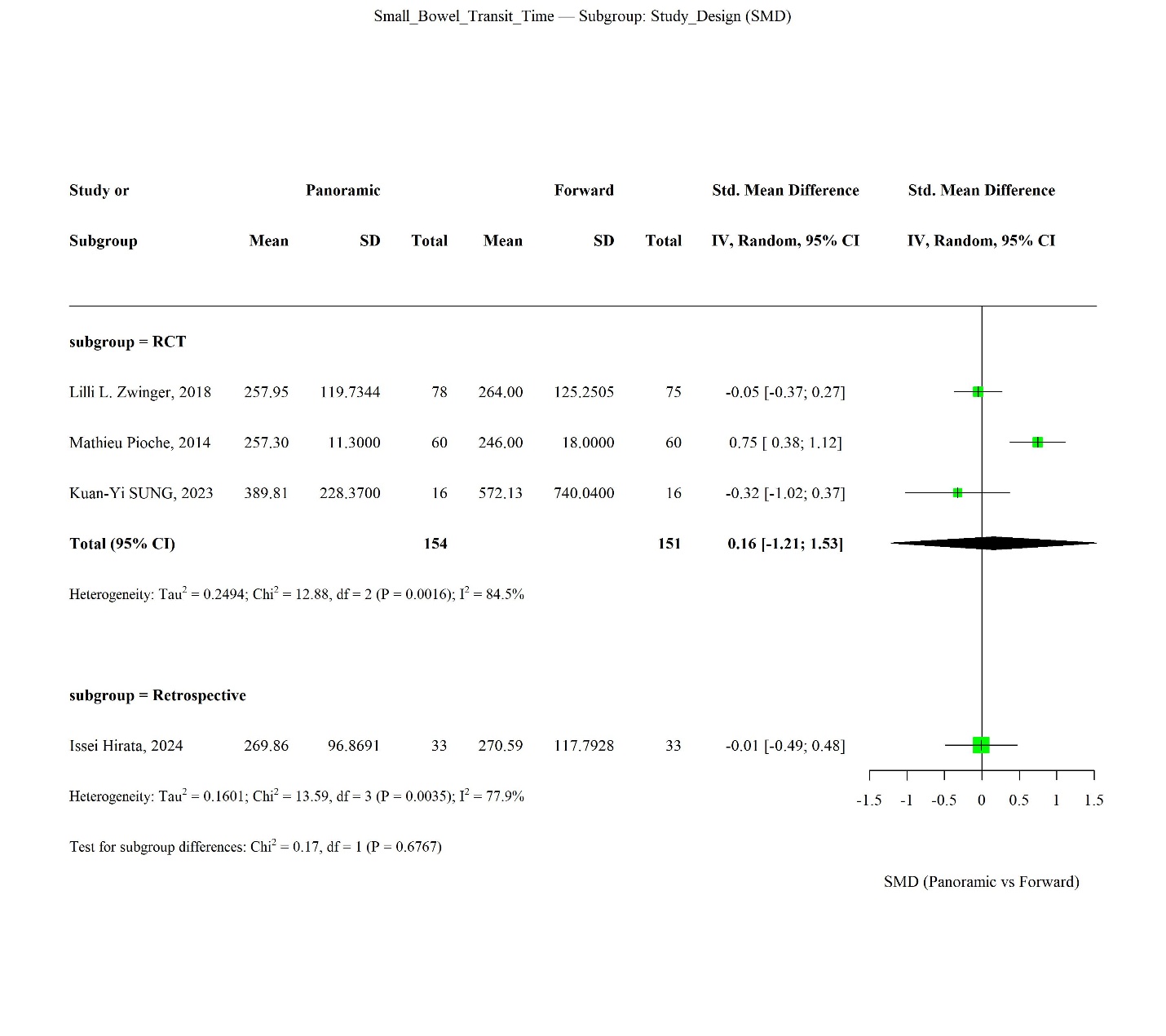


**Supplementary Figure 24.** Forest plot of subgroup analysis by study design for the gastric transit time of capsule endoscopy. SMD, Standardized Mean Difference; SD, Standard Deviation; CI, confidence interval; IV, Inverse variance.


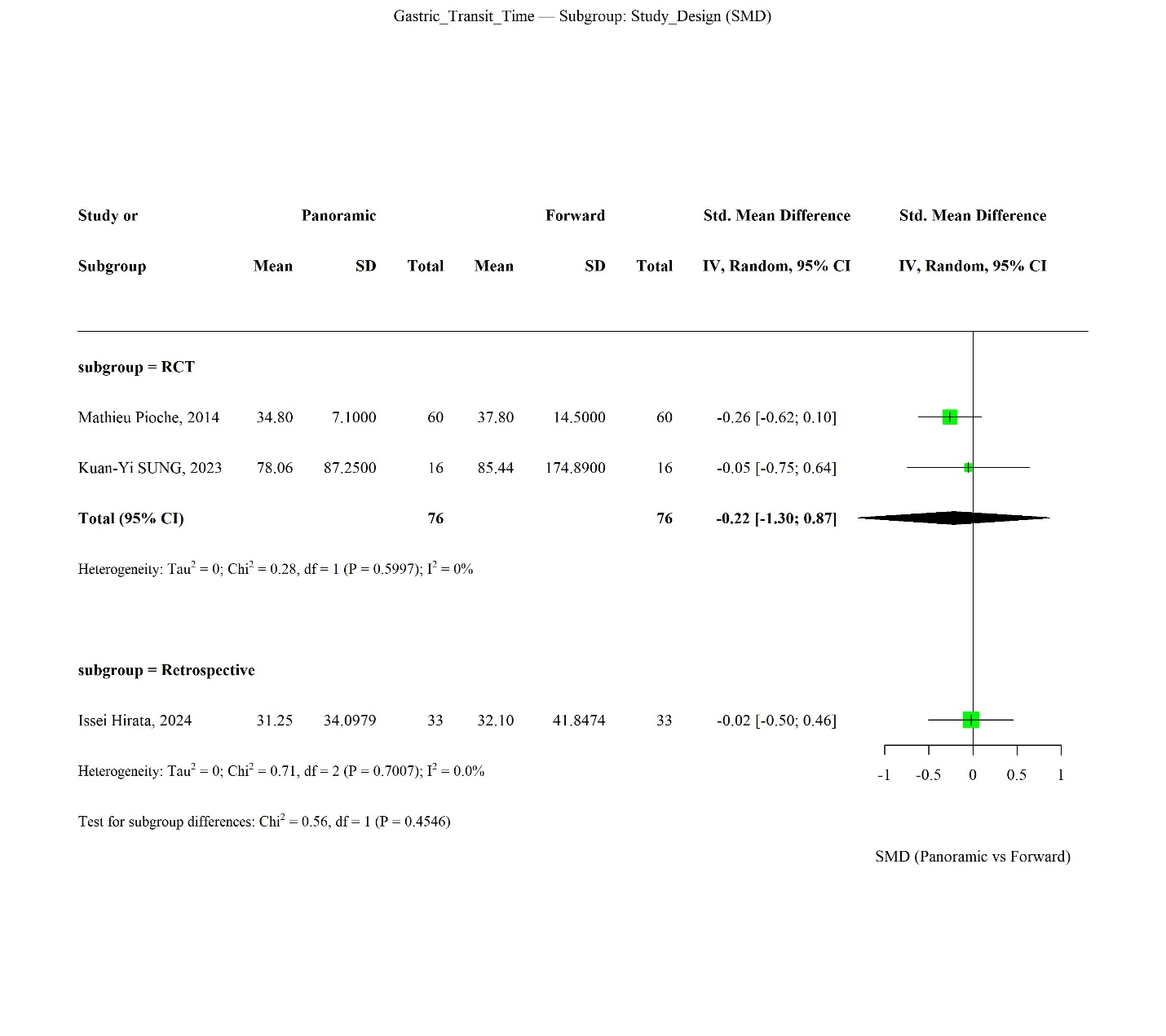


**Supplementary Figure 25.** Forest plot of subgroup analysis by study design for the reading time of capsule endoscopy. SMD, Standardized Mean Difference; SD, Standard Deviation; CI, confidence interval; IV, Inverse variance.


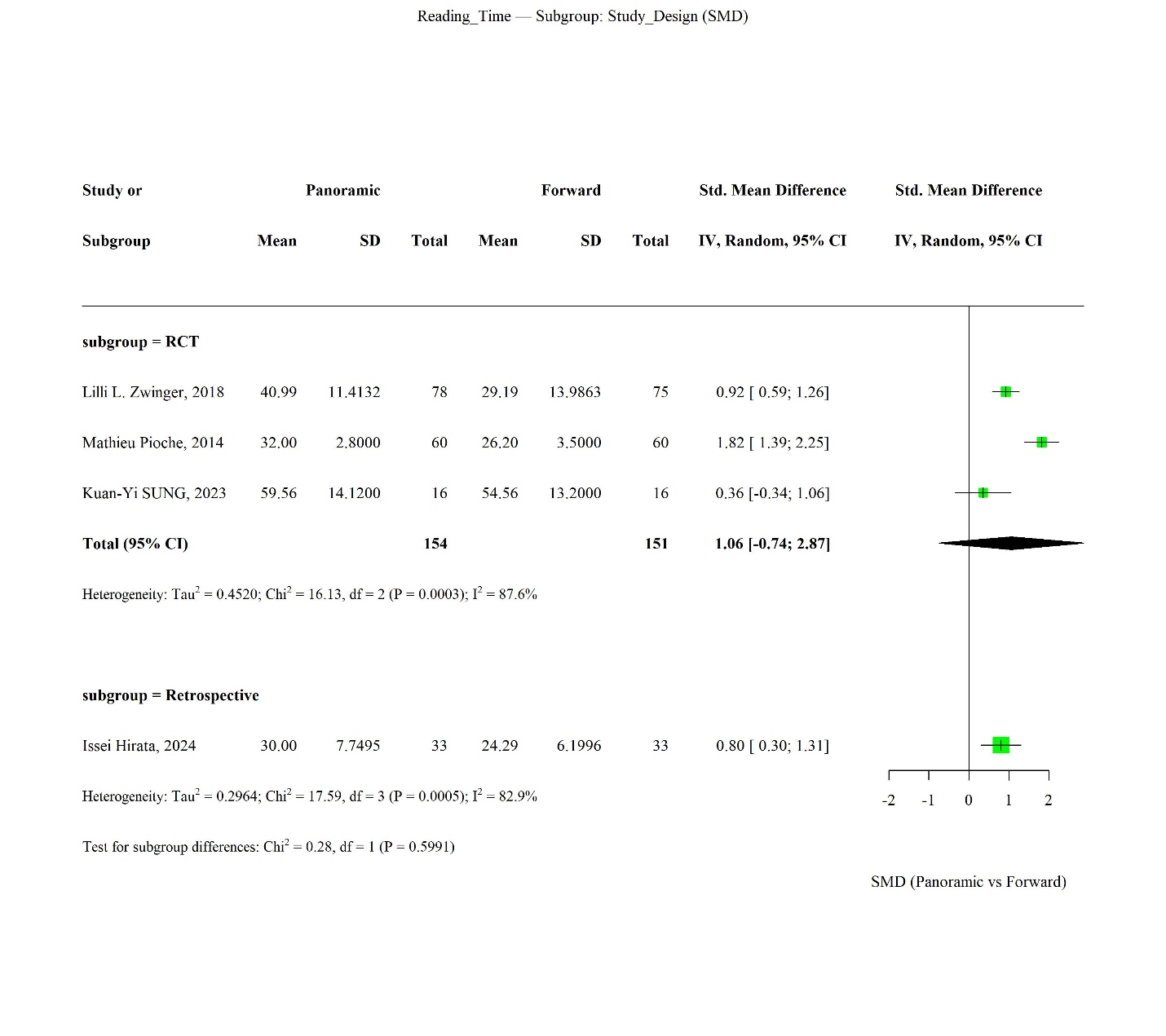


**Supplementary Figure 26.** Forest plot of subgroup analysis by study design for the completion of capsule endoscopy. RD, risk difference; MH, Mantel-Haenszel

CI, confidence interval.


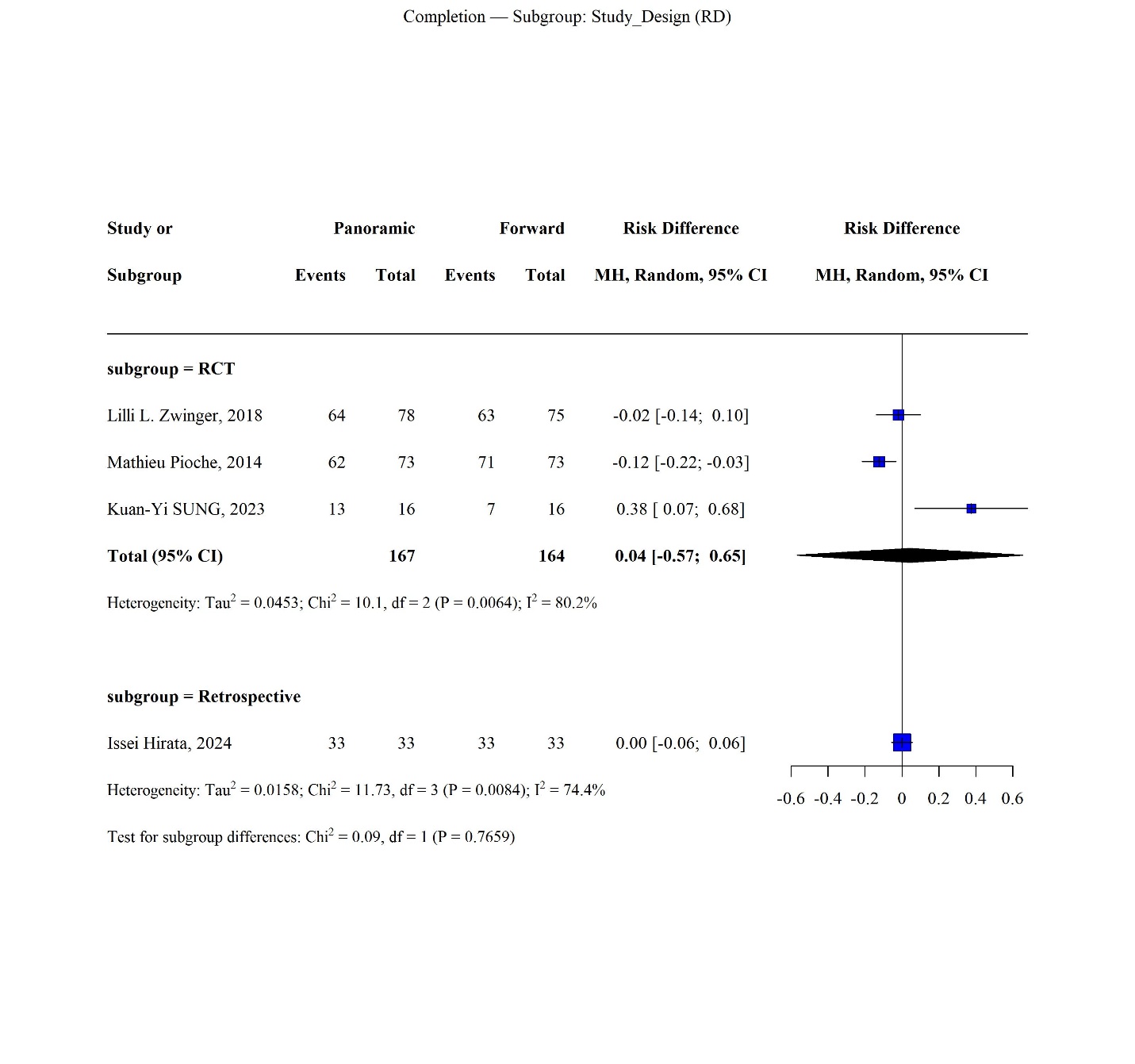


**Supplementary Figure 27.** Forest plot of subgroup analysis by study design for the technical fault of capsule endoscopy. RD, risk difference; MH, Mantel-Haenszel

CI, confidence interval.


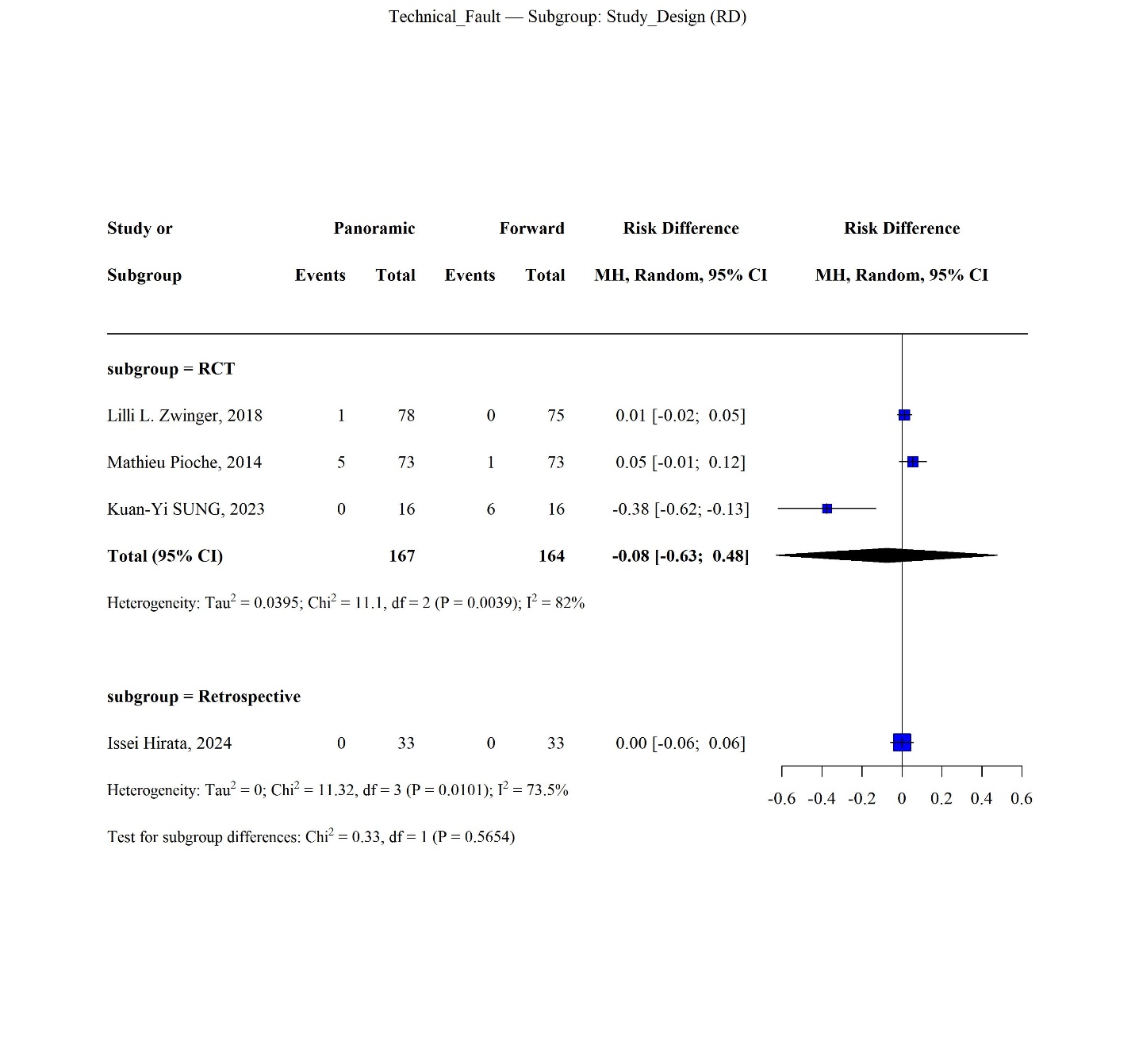


**Supplementary Figure 28.** Forest plot of subgroup analysis by study design for the retention of capsule endoscopy. RD, risk difference; MH, Mantel-Haenszel

CI, confidence interval.


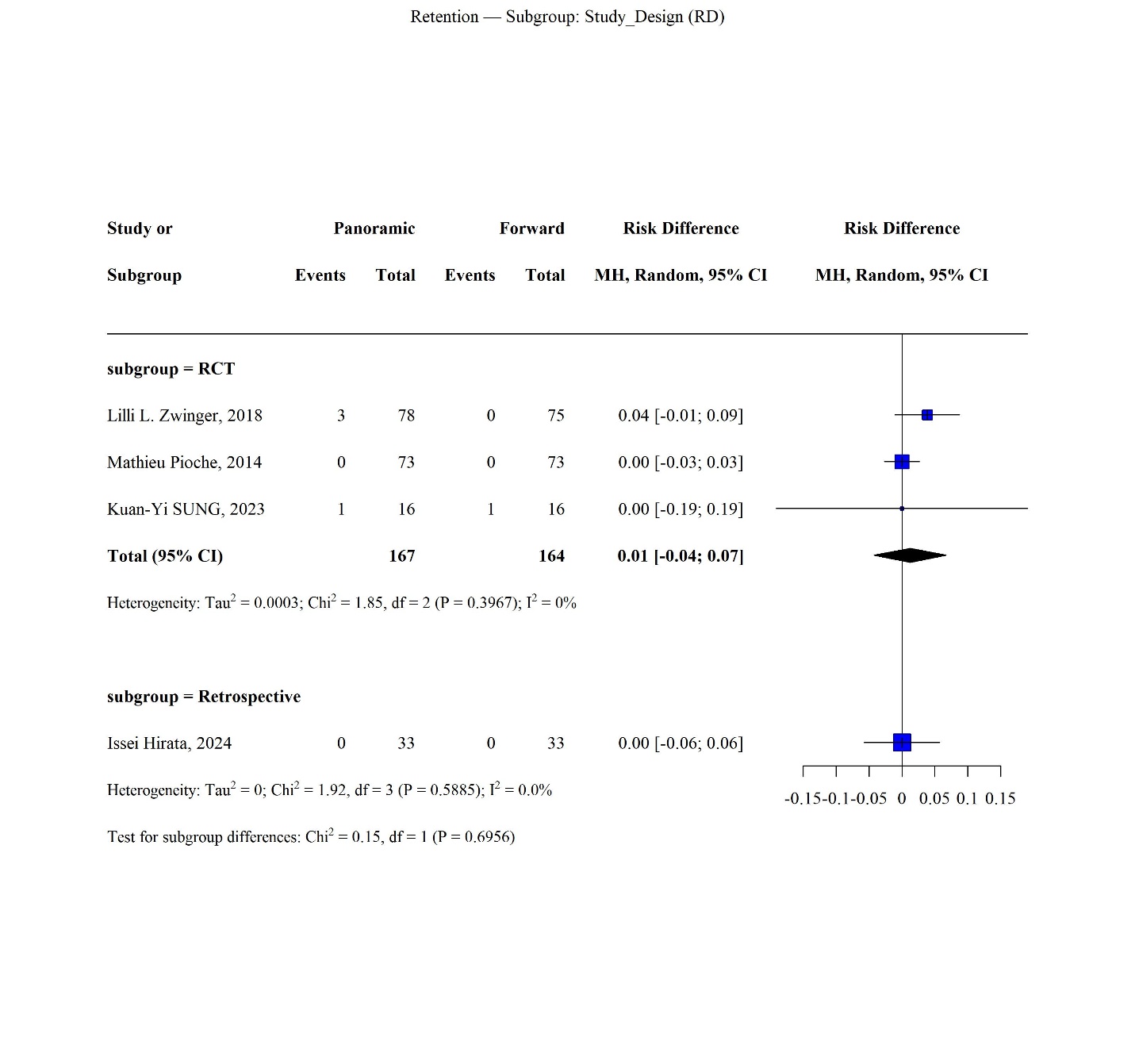


**Supplementary Figure 29.** Forest plot of subgroup analysis by study design for the incomplete transit of capsule endoscopy. RD, risk difference; MH, Mantel-Haenszel

CI, confidence interval.


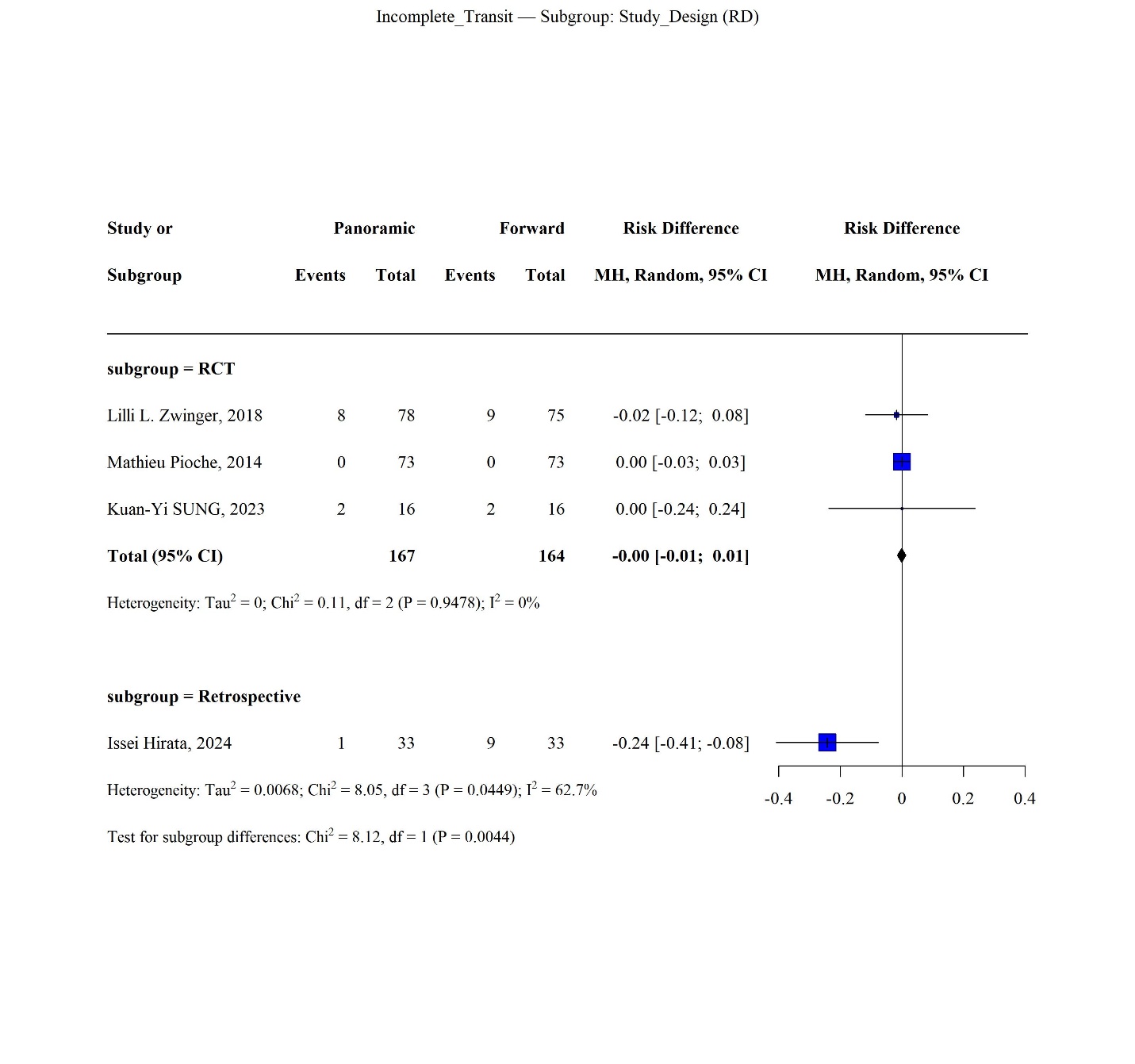


**Supplementary Figure 30.** Forest plot of subgroup analysis by study design for the insufficient preparation of capsule endoscopy. RD, risk difference; MH, Mantel-Haenszel

CI, confidence interval.


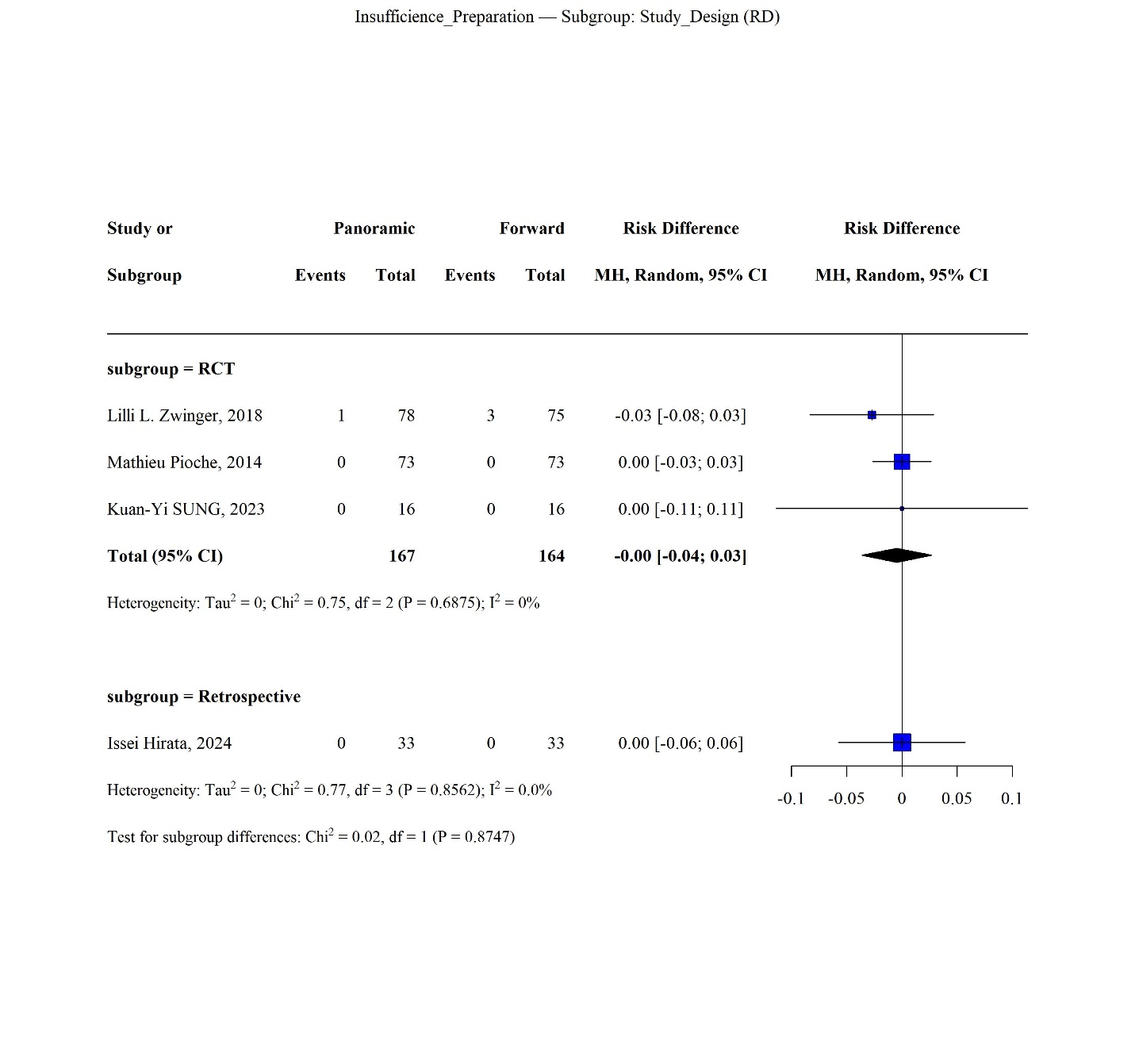

Supplement: Supplementary file 1 — Supporting File [file HSR2-9-e72095-s001.docx]
